# TRPA1 promotes overactive bladder progression by activating the NLRP3 inflammasome and driving pyroptosis

**DOI:** 10.1038/s41419-026-08426-5

**Published:** 2026-02-16

**Authors:** Yongjuan Rao, Yunran Wang, Jie Gao, Xun Guan, Sixuan Lv, Junlian Gu, Kefang Wang

**Affiliations:** 1 School of Nursing and Rehabilitation, Cheeloo College of Medicine, Shandong University, Jinan, Shandong China; 2 Department of Nursing, The First Affiliated Hospital of Nanchang University, Nanchang, Jiangxi China

**Keywords:** Protein-protein interaction networks, Cell death, Transcriptomics

## Abstract

Overactive bladder (OAB) is strongly linked to intravesical inflammatory responses. Transient receptor potential ankyrin-1 (TRPA1) is a key sensor and signaling molecule in bladder function, crucial for initiating and maintaining inflammation. However, the mechanisms underlying TRPA1-mediated inflammatory processes in OAB remain unclear. This study aims to elucidate the molecular mechanisms of TRPA1 and the contribution of inflammatory pathways to OAB. Elevated TRPA1 expression was observed in urinary sediment cells from OAB patients and in bladder tissues from OAB animal models. Mechanistically, TRPA1 drives the progression of OAB by activating the Nod-Like Receptor Family Pyrin Domain Containing 3 (NLRP3) inflammasome and triggering pyroptosis. Notably, treatment with HC-030031 effectively mitigated inflammatory responses and restored bladder function, whereas *Nlrp3* overexpression negated these therapeutic benefits. Furthermore, TRPA1-mediated upregulation of NLRP3 was dependent on the transcription factors MAZ and SMAD3, highlighting a novel regulatory axis. Our findings establish TRPA1 as a pivotal mediator in the progression of OAB by activating the NLRP3 inflammasome and thereby inducing pyroptosis. Targeting TRPA1 or the NLRP3 signaling pathway represents a promising therapeutic strategy for OAB, offering new insights into disease management and potential improvements in patient outcomes.

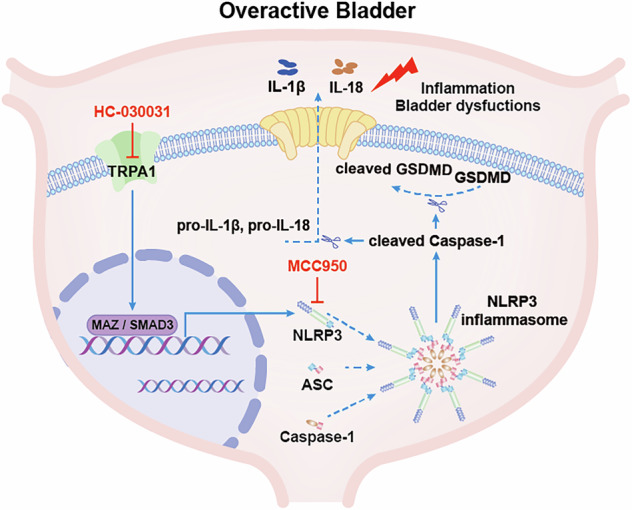

## Introduction

Overactive bladder (OAB) is a prevalent urological disorder characterized by urgency, frequent urination, and increased nocturia, with or without urgency urinary incontinence. It affects approximately 20% of the global population, significantly disrupting individuals’ daily urination patterns and overall quality of life [1]. Despite the considerable prevalence and significant impact of OAB, current therapeutic management approaches exhibit limited efficacy. Available pharmacological treatments for OAB typically offer only modest improvements relative to placebo. Moreover, the side effects associated with these medications, coupled with their limited efficacy, significantly contribute to poor patient adherence [2–4]. This challenge largely stems from a failure to adequately target the molecular mechanisms that underpin the pathophysiology of OAB.

Inflammation is a key characteristic of various bladder dysfunctions and plays a crucial role in influencing the urination reflex and overall bladder health [5, 6]. Prior studies have shown that patients with OAB exhibit elevated levels of inflammatory markers, including IL-6 and C-reactive protein in both urine and serum sample [7, 8]. However, the precise mechanisms by which inflammation participates in OAB pathogenesis remain poorly defined, necessitating further exploration. Pyroptosis, a gasdermin-mediated inflammatory form of programmed cell death, is classically initiated through inflammasome activation and downstream signaling cascades. Upon stimulation by diverse activating signals, intracellular pattern recognition receptors such as the Nod-Like Receptor Family Pyrin Domain Containing 3 (NLRP3) undergo oligomerization to recruit apoptosis-associated speck-like protein containing a CARD (ASC) and pro-cysteinyl aspartate specific proteinase-1 (pro-Caspase-1), thereby assembling active inflammasome complexes and activating Caspase-1. Activated Caspase-1 subsequently cleaves full-length Gasdermin D (GSDMD) and processes pro-IL-1β and pro-IL-18 into mature forms. This cascade culminates in the release of IL-1β, IL-18, and other cellular contents through gasdermin pores, triggering pyroptosis and inflammatory responses [9]. Existing evidence implicates that GSDMD-mediated pyroptosis exacerbates cisplatin-induced inflammatory responses and kidney injury [10], and promotes pulmonary inflammation and fibrosis progression [11]. However, whether pyroptosis-mediated inflammation contributes to the progression of OAB remains unexplored and requires further elucidation.

Transient receptor potential (TRP) channels are integral membrane proteins that function as ion channels, playing a vital role in numerous physiological processes [12]. Among these, transient receptor potential ankyrin-1 (TRPA1), a polymodal ion channel activated by diverse physical and chemical stimuli [13], plays a pivotal role in inflammation and tissue injury, and has been recognized as a gatekeeper of inflammation [14]. Recent research has also highlighted the significant role of TRPA1 in triggering inflammatory responses. In cardiomyocytes, the application of TRPA1 inhibitors has been shown to diminish oxidative stress, inflammation and apoptosis, lower the level of pro-inflammatory cytokines, including IL-1β, IL-6, IL-17 and TNF-α [15]. Similarly, in airway epithelial cells, TRPA1 inhibitors attenuate cigarette smoke-induced elevations in mRNA levels of pro-inflammatory cytokines (e.g., *Il1b* and *Il18*) and protein expression of NLRP3 and Caspase-1 [16]. These findings collectively suggest a potential role of TRPA1 in modulating pyroptosis and inflammatory cascades. However, the precise mechanistic contributions of TRPA1 to OAB pathogenesis, particularly its interplay with pyroptosis and inflammation within this pathological context, remain to be fully elucidated.

In this study, we explored the role of TRPA1 in OAB progress. We observed significantly elevated TRPA1 expression in urinary sediment cells from clinical OAB patients and bladder tissues of OAB animal models. We demonstrated that inhibiting TRPA1, both in vivo and in vitro, with the TRPA1 inhibitor HC-030031, provides therapeutic benefits against inflammatory responses and symptoms linked to OAB. Specifically, pharmacological inhibition of TRPA1 mitigated pyroptosis and inflammatory responses while ameliorating bladder function in OAB model mice through suppression of NLRP3 inflammasome activity. Conversely, *Nlrp3* overexpression in the bladder abolished these therapeutic benefits. Further investigations have delineated MAZ and SMAD3 as key downstream transcription factors modulated by TRPA1, underscoring their pivotal roles in NLRP3 inflammasome activation. These discoveries illuminate a novel function for TRPA1 in the pathogenesis of OAB, emphasizing its potential as a therapeutic target for managing this disorder. Moreover, they provide fresh insights into the intricate interplay among TRPA1, pyroptosis, and inflammatory cascades, potentially paving the way for innovative interventions to alleviate OAB symptoms and address its underlying pathophysiology.

## Methods

### Animals and models

Female C57BL/6J mice aged 7 weeks and female Sprague-Dawley rats aged 7 weeks were provided by Beijing Vital River Laboratory Animal Technology Co., Ltd. In this study, we employed three batches of distinct animal models, which are described as follows:

Seven-week-old female C57BL/6J mice were randomly assigned to two groups (5/6 mice per group): (1) Control (Ctrl) group; (2) Cyclophosphamide (CYP) group. To establish the short-term CYP-induced OAB mouse model, intraperitoneal injections of CYP (80 mg/kg; Sigma, USA) were administered every other day for a total of four injections over 7 days. The CYP was dissolved in 0.9% NaCl (Shandong Qidu Pharmaceutical Co., Ltd., Shandong, China), while the Ctrl mice received only an equal volume of 0.9% NaCl. The rats were randomly assigned to two groups (5/6 rats per group): (1) Sham group; (2) Partial bladder outlet obstruction (pBOO) group. To construct long-term pBOO-induced OAB rat model, the proximal urethra was exposed after the bladder was found. Then, introduced a urethral catheter (outer diameter of 1.2 mm) into the bladder, ligated the proximal urethra with 4-0 silk (Yangzhou Goldenwell Medical Devices Factory, Jiangsu, China). Finally, closed the incision layer by layer after removing the catheter. The Sham group underwent the same procedure without urethral ligation.

Seven-week-old female C57BL/6J mice were randomly assigned to four groups (5/6 mice per group): (1) Ctrl group; (2) CYP group; (3) HC-030031 (HC) group; (4) CYP + HC group. On the eighth day, the HC group and CYP + HC group received intraperitoneal injections of HC-030031 (100 mg/kg, MedChemExpress, USA), whereas other mice continued to receive an equivalent volume of 0.9% NaCl.

Seven-week-old female C57BL/6J mice were randomly assigned to three groups (5/6 mice per group): (1) CYP group; (2) CYP + HC group, (3) CYP+HC+adeno-associated virus type 9 encoding *Nlrp3* (AAV9-*Nlrp3*) group. To establish the *Nlrp3* overexpression model, AAV9-*Nlrp3* was injected into the bladder of the mice after one week of adaptive feeding, with a virus dose of 1 × 10^11^ vg/ mouse. The other two groups received an equivalent volume of an empty vector. After a 2-week period post-viral injection to establish stable expression, the mice were treated with CYP and HC-030031 as previously described to construct the corresponding mouse model. The AAV9 and the overexpression vector for *Nlrp3* were procured from He Yuan Biotechnology Co., Ltd. in Shanghai.

### Data and urine collection from clinical subjects

This study recruited female nurses from a tertiary hospital in Jinan, Shandong Province, who had been employed for at least one year and were willing to participate. Exclusions included nursing students, pregnant nurses, and those with severe lower urinary tract disorders or recent pelvic surgeries. Clinical data collected encompassed age, body mass index, chronic constipation, maternal history, menstrual irregularities, and Overactive Bladder Symptom Score (OABSS). OAB is diagnosed with a total OABSS ≥ 3 and the urgency score ≥2. Midstream urine samples (more than 50 mL) were collected from the first morning void. Urine was transported at low temperatures, centrifuged at 2300 rpm for 5 min, followed by a second centrifugation at 10,000 rpm for urine sediment cells collection.

### Cell culture and treatments

The human bladder cancer cell line 5637 was cultivated in RPMI-1640 medium (Gibco, Grand Island, NY, USA) supplemented with 10% fetal bovine serum (Gibco), 100 U/mL penicillin (Biosharp, Anhui, China), and 100 µg/mL streptomycin (Gibco). To investigate the effects of various treatments, 5637 cells were exposed for 24 h to Acrolein (AC, 15 µM, Aladdin), HC-030031 (1 mM), MCC950 (10 µM, Aladdin, China), and AITC (30 µM, MedChemExpress). Following exposure, cells were harvested for subsequent assays. The 5637 cell line utilized in this study was obtained from the ATCC Cell Bank.

### Void spot assay

Filter paper (Cytiva, UK) devoid of autofluorescence was used to completely cover the bottom of the cage, where each mouse was gently placed for individual observation over a 4-h period. Following this, the filter paper was removed, dried, and exposed to ultraviolet light in a dark environment to capture images. ImageJ software was employed to manually delineate the outlines of urine diffusion, allowing for the quantification of the total number of urine spots and the corresponding area of each spot. A standard curve of urine volume versus spot area was generated by applying 20, 30, 50, 70, 100, 150, and 200 µL of mouse urine onto filter paper. The total urine volume was then calculated based on the urine area-to-volume ratio.

### Urodynamic test

Mice and rats were anaesthetized with intraperitoneal 5% urethane (1 g/kg, Sigma), and the bladders were exposed. A 20 mL Luer interface syringe containing 0.9% NaCl was mounted on a microsyringe pump (KD Scientific, USA). The pressure sensor (ADInstruments, Australia) was calibrated after connecting to a three-way connector. A PE-50 catheter (SDR Scientific, Australia) was inserted into the bladder and secured with 5-0 silk thread, linking the other end to the syringe and pressure transducer. Prewarmed 0.9% NaCl was infused at 0.006 mL/min for mice and 0.02 mL/min for rats. The PowerLab system recorded micturition waveforms and various bladder pressure parameters.

### RNA-sequencing (RNA-seq)

The bladder samples were frozen on dry ice and transported to Shanghai Meiji Biomedical Technology Co., Ltd. for RNA-seq sequencing analysis. The number of read counts of each gene was obtained by FeatureCounts, and DESeq2 was used to perform differential expression analysis. Genes with an adjusted *P*-value less than 0.05 and an absolute log2 fold-change value greater than 1 were considered differentially expressed compared to the reference genome. Finally, Gene Set Enrichment Analysis (GSEA) and Kyoto Encyclopedia of Genes and Genomes (KEGG) pathway enrichment analysis were performed.

The interpretation of GSEA results hinges on several key features. The Normalized Enrichment Score (NES) reflects the strength and direction of enrichment of the gene set at the top or bottom of the gene list ranked by their correlation with the phenotype. A larger absolute NES value indicates stronger enrichment, with a positive NES suggesting overall upregulation of the pathway, and a negative NES indicating overall downregulation. The False Discovery Rate (FDR), a core metric adjusted for multiple hypothesis testing that evaluates the significance of the NES, representing the probability of a false positive enrichment result, where an FDR < 0.25 is generally considered statistically significant. And the *P*-value, which reflects the raw significance of the enrichment score but should be interpreted with the FDR as the primary reference for final conclusions.

In KEGG pathway enrichment analysis, the results are gauged by the GeneRatio, Q-value, and count of enriched genes. The x-axis depicts the GeneRatio, with higher values reflecting greater enrichment. The y-axis displays significantly enriched pathway terms. The Q-value, an adjusted *P*-value for multiple comparisons, is encoded by color intensity on a logarithmic scale, where deeper red hues denote more significant enrichment. Dot size corresponds to the number of differentially expressed genes in each term, with larger dots representing more genes.

### Real-time quantitative polymerase chain reaction (RT-qPCR)

Total RNA was extracted from tissue and cell samples using TRIzol reagent (Cwbio, Valencia, CA, USA). cDNA synthesis was performed with the HiFiScript cDNA Synthesis Kit (Cwbio, USA). RT-qPCR was conducted with UltraSYBR Mixture (Cwbio, USA) in a Roche LightCycler 480 (Roche, Germany).

### Western blot analysis

Protein samples were lysed in cold RIPA lysis buffer (Beyotime Biotechnology, Shanghai, China) supplemented with a cocktail of protease and phosphatase inhibitors (Beyotime Biotechnology). The supernatant from the tissue or cell lysates was obtained by centrifugation at 4 °C for 20 min at 12,000 rpm. Proteins were then separated via electrophoresis using 10% or 12% SDS-PAGE gels and transferred onto nitrocellulose membranes (GE Healthcare Life Sciences, Beijing, China) according to standard protocols. Following a 2-h blocking step with 5% skim milk (BD Biosciences, USA) at room temperature, the nitrocellulose membranes were incubated overnight at 4 °C with appropriate primary antibodies. The primary antibodies we used were anti-TRPA1 (1:500, Novus biologicals, USA and 1:1000, Proteintech, USA), anti-NLRP3 (1:1000, Cell Signaling Technology, USA), anti-Caspase-1 (1:5000, Proteintech, USA), anti-Cleaved Caspase-1 (1:1000, Proteintech, USA), anti-Gasdermin-D (1:1000, Abclonal, China), anti-Cleaved Gasdermin-D (1:1000, Abclonal, China), and anti-β-actin (1:1000, Servicebio Technology, China). Then the membranes were reacted with appropriate horseradish peroxidase (HRP)-conjugated secondary antibody (1:8000, Jackson ImmunoResearch, USA) at room temperature for 1.5 h, and the immune complexes were visualized using enhanced chemiluminescence reagents.

Protein bands were quantified using ImageJ software. Original images were converted to 8-bit grayscale and inverted. Each band was then delineated, and its raw integrated density value (RawIntDen) was measured. Ratios of the target protein to the internal control protein were calculated and normalized to the mean value of the control group. These normalized data were subsequently employed for statistical analysis.

### Histological staining

The bladder tissue was cut into 4 μm sections. To detect structural changes in the bladder tissue, the sections were stained by Hematoxylin-Eosin Kit (H&E, Solarbio Life Sciences, China). Masson’s trichrome staining kit (Solarbio Life Sciences, China) was used to visualize collagen fibers, muscle fibers and other tissue structures in the bladder. The expression of TRPA1 in the bladder of mice was detected by Immunohistochemistry (IHC) staining. After treatment of antigen retrieval, add 3% hydrogen peroxide (Sigma, USA) solution to the bladder sections (25 min, room temperature), blocked (3% BSA, Solarbio Life Sciences, China, 30 min, room temperature) and incubated overnight at 4 °C with the primary antibody against TRPA1 (1:200, Novus Biologicals, USA). Then the bladder sections were incubated with a biotinylated secondary antibody (1:1000, Abcam, UK, 1.5 h, room temperature). Finally, using Diaminobenzidine (DAB) Histochemistry Kit (Solarbio Life Sciences) and hematoxylin (Solarbio Life Sciences) to stain bladder section slides. The localization and expression of NLRP3 were detected by Immunofluorescence (IF) staining. Primary antibody was anti-NLRP3 (1:200, Cell Signaling Technology, USA). Fluorescent secondary antibody (1:1000, Abcam, UK) was used for incubation, and DAPI staining solution (Beyotime Biotechnology, China) was used for nuclear staining. Sections images obtained with light microscopy (Nikon, Tokyo, Japan) or fluorescence microscope (Nikon).

The IHC images underwent quantitative analysis using ImageJ software. Following conversion to 8-bit grayscale, a threshold was applied to delineate the positively stained regions, and the percentage of the positive area was computed. These percentages were normalized and subjected to statistical analysis. The same quantification approach was applied to Masson’s trichrome staining.

### Immunofluorescence co-localization

Immunofluorescence co-localization was used to detect the co-localization of TRPA1, Na/K-ATPase, and the expression of TRPA1 on the cell membrane. The reagents used include: phosphate-buffered solution (PBS, Solarbio Life Sciences, China), 4% ice methanol (Fuyu Fine Chemical Co., Ltd.), 0.5%Triton (Beyotime Biotechnology), 3% BSA (Solarbio Life Sciences), DAPI (Beyotime Biotechnology), Antifade Mounting Medium (Beyotime Biotechnology). Line scan of immunofluorescence images with ImageJ reveals changes in protein fluorescence intensity.

### Cell viability/cytotoxicity assays

For the CCK8-assay, add 10 μL CCK-8 solution (Beyotime Biotechnology, China) to each well and incubate the plate in the incubator for 1–4 h. Cell viability was analyzed by measuring the absorbance at 450 nm using the microplate reader. To assess the relative cell number, the total Lactate Dehydrogenase (LDH) activity was quantified from cell lysates using a Lactate Dehydrogenase Kit (Solarbio Life Sciences, China). Live and dead cells were simultaneously distinguished using Calcein-/PI Cell Viability/Cytotoxicity Assay Kit (Beyotime Biotechnology, China).

### Dual luciferase reporter assay

To investigate the regulatory effects of TRPA1 on NLRP3 promoter activity, after the cells were co-transfected with Firefly luciferase reporter plasmids encoding *NLRP3* promoter (empty vector, pGL3-*NLRP3*-promoter WT, pGL3-*NLRP3*-promoter MUT1, pGL3-*NLRP3*-promoter MUT2, pGL3-*NLRP3*-promoter MUT3), *TRPA1* overexpressed plasmid and Renilla luciferase reporter plasmids for 48 h, cells then lysed by Passive Lysis Buffer (Dual-Luciferase® Reporter Assay System, Promega, USA). The luciferase activity was measured by a dual luciferase reporter assay system. The relative firefly luciferase activity was normalized to Renilla luciferase activity.

### Chromatin immunoprecipitation (ChIP) assay

ChIP assays were performed following the standard procedure using SimpleChIP Enzymatic Chromatin IP Kit (Cell Signaling Technology, USA). Briefly, treated cells were cross-linked with 1% formaldehyde, and the prepared chromatin was then immunoprecipitated with anti-MAZ (1:50, Active Motif, China), anti-SMAD3 (1:50, Cell Signaling Technology, USA) and anti-IgG (1:1000, Abcam, UK) at 4 °C overnight. Immunoprecipitated DNA was subjected to real-time PCR analysis.

### Co-Immunoprecipitation (Co-IP) assay

For the Co-IP assay, cells were harvested and lysed with IP buffer containing protease and phosphatase inhibitors. After centrifugation, the supernatant was incubated with an anti-TRPA1 antibody for 2 hours before the addition of Protein A/G PLUS-Agarose beads (Santa Cruz Biotechnology, USA) and subsequent overnight incubation at 4 °C. The immunoprecipitation samples were verified by western blot with anti-MAZ (1:100, Active Motif, China), anti-SMAD3 (1:100, Cell Signaling Technology, USA).

### Statistical analysis

Data visualization and statistical analyses were performed using GraphPad Prism 9. Statistical significance was evaluated using unpaired two-sided *t* test for two-group comparisons or one-way analysis of variance (ANOVA) for multiple groups, followed by Tukey’s post hoc test for pairwise comparisons. Correlations were analyzed using nonparametric Spearman correlation. A *P*-value of <0.05 (*P < 0.05) was considered statistically significant. All experiments were performed in triplicate unless otherwise noted.

### Ethics approval and consent to participate

All procedures were performed in compliance with relevant laws. Informed consent was obtained from the clinical subjects before collecting the questionnaires and urine samples, and the experimental design was approved by the Ethics Committee of the School of Nursing and Rehabilitation of Shandong University (permit date: September 12, 2021, permit number: 2021-R-135). All animal protocols and experiments were approved by the Ethics Committee of School of Nursing and Rehabilitation of Shandong University (permit date: March 1, 2022, permit number: 2022-R-117). The Ethics Committee of School of Nursing and Rehabilitation of Shandong University adheres to the principles and guidelines set forth by the International Council for Laboratory Animal Science (ICLAS), ensuring that our animal research meets international ethical standards.

## Results

### Upregulation of TRPA1 in bladder tissues from preclinical OAB models and in urine sediment cells from clinical OAB patients

**Fig. 1:**
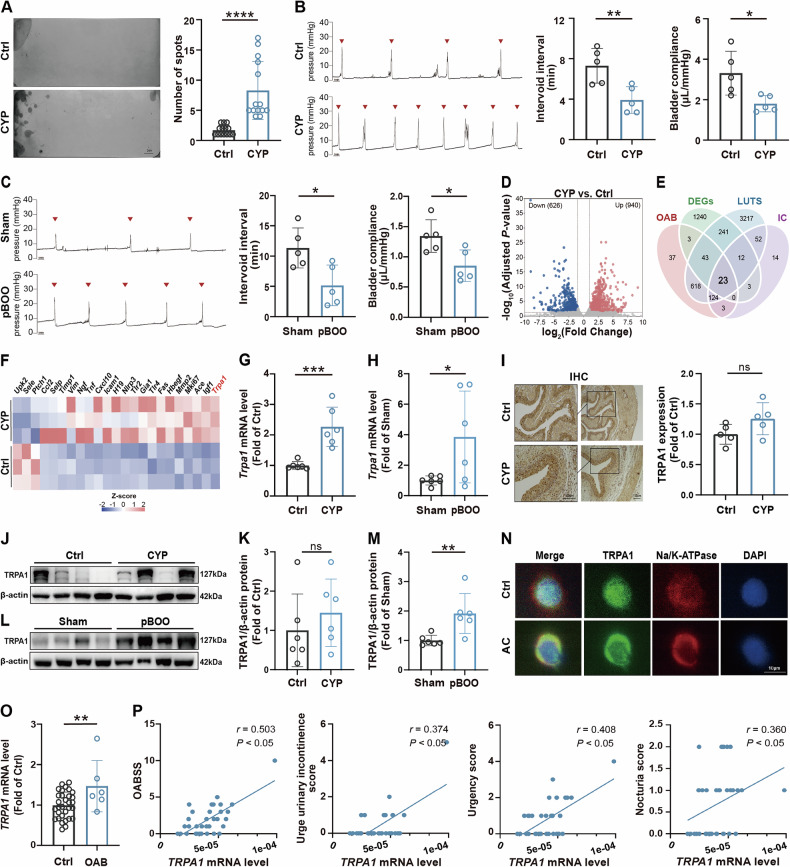
Upregulated TRPA1 in OAB models. **A** Void spot assay was utilized to assess urination behavior, specifically measuring the frequency of urine points in mice (n = 14). **B** Urodynamic test was conducted to monitor key bladder parameters, including intervoid intervals and bladder compliance in mice (*n* = 5). **C** Urodynamic test in rats to monitor key bladder parameters (*n* = 5). **D** The volcano plot illustrates the DEGs in the bladders of Ctrl and CYP-induced OAB mice. **E** The Venn diagram depicts the overlap of genes associated with OAB, LUTS, and IC, as well as DEGs in our RNA-seq analysis. **F** Heatmap showcases the expression of common genes obtained from **E** in Ctrl and CYP-induced OAB mice. Colors represent Z-score of normalized expression. **G**, **H** RT-qPCR analyses were employed to quantify *Trpa1* mRNA levels in the bladder tissues of mice and rats, respectively (*n* = 6). **I** IHC staining was utilized to assess TRPA1 expression in bladder tissue of mice. **J–M** Western blot analysis quantified the protein levels of TRPA1 in the bladders of both mice and rats (*n* = 6). **N** Immunofluorescence co-localization assay identified TRPA1 localization at the plasma membrane of 5637 cells, where Na/K-ATPase staining (plasma membrane) is shown in red, TRPA1 in green, and DAPI (nuclei) in blue. **O** RT-qPCR was utilized to determine *TRPA1* mRNA levels in human urine sediment cells. **P** The correlations between *TRPA1* mRNA levels and both the total OABSS and its individual components (including urge urinary incontinence, urgency, and nocturia scores) were analyzed using nonparametric Spearman’s correlation. Data are presented as mean ± SD. *P < 0.05, **P < 0.01, ***P < 0.001, ****P < 0.0001 or ns (not significant) using unpaired *t* test.

The short-term OAB mouse model was successfully constructed by repeated injections of low dosages of CYP [17–19], as determined by impaired bladder function. Void spot assay showed that the CYP mice had a significantly greater number of urinary spots (Fig. 1A), but had no difference in total volume of urine with the Ctrl mice (Fig. S1A), indicating a hyperactive voiding characteristic of the CYP mice. Urodynamic test similarly showed that the CYP mice had an increased voiding frequency, and a significant increase in resting pressure and threshold pressure (Fig. 1B and Fig. S1B, C), but a decrease in bladder compliance (Fig. 1B), which suggested that the CYP mice were more sensitive to bladder filling and needed less urine to trigger voiding. No changes were observed for pressure amplitude or peak pressures (Fig. S1D, E). In addition, the lamina propria of bladder slices in the CYP mice showed significant edema (Fig. S1F), which was also evidenced by the increase in bladder weight and the ratio of bladder weight to body weight (Fig. S1G, H). Whereas no significant areas of fibrosis occurred in the muscular layer (Fig. S1I).

We subsequently induced a long-term OAB rat model using the pBOO surgery [20]. Consistent with our expectations, the pBOO rats also displayed an OAB phenotype (Fig. 1C and Fig. S1J-M). The pBOO rats exhibited edema in the interstitial layer (Fig. S1N) and a slight increase in bladder weight and bladder weight ratio compared to the Sham group, although these changes did not reach statistical significance (Fig. S1O, P). Additionally, the bladder wall of the pBOO rats demonstrated pronounced fibrosis (Fig. S1Q).

To investigate the genes and signaling pathways relevant to OAB, we conducted RNA sequencing on bladder samples from the CYP mice, identifying differentially expressed genes (DEGs) (Fig. 1D). From GeneCards, we compiled a list of genes closely associated with bladder dysfunction, specifically those linked to OAB, lower urinary tract symptoms (LUTS), and interstitial cystitis (IC). Our analysis revealed a significant overlap between the genes associated with OAB, LUTS, and IC, and the DEGs identified in our study (Fig. 1E). As illustrated by the heatmap of shared genes (Fig. 1F), *Trpa1* mRNA level exhibited a significant increase in the CYP mice. Correspondingly, RT-qPCR analyses revealed that *Trpa1* mRNA levels were markedly elevated in both CYP mice and pBOO rats (Fig. 1G, H). Consistently, we retrieved data from the PRJEB24781 database, which indicated that *Trpa1* mRNA level in urinary bladder smooth muscle cells was significantly increased under a TNF-α-induced microenvironment associated with bladder dysfunction (Fig. S1R). To further investigate the expression of TRPA1 in the context of OAB, we assessed the protein level of TRPA1 in CYP mice bladder tissue. Notably, both IHC staining and Western blot analysis revealed a trend toward increased total TRPA1 protein levels in the bladder tissue of CYP group compared to the Ctrl group. However, this difference did not achieve statistical significance (Fig. 1I-K), which aligns with previous findings [21]. In contrast, we observed a significant increase in TRPA1 protein levels in the bladder tissue of pBOO rats (Fig. 1L, M). These findings highlight a differential expression of TRPA1 in various models of bladder dysfunction, suggesting distinct pathophysiological mechanisms involved in CYP and pBOO.

Given that TRPA1 is a transmembrane protein, its expression on the plasma membrane is crucial for modulating TRPA1 channel activity [22–25]. To assess the localization of TRPA1 in the plasma membrane, we exposed the human bladder cancer cell line 5637 to Acrolein (AC), a CYP metabolite implicated in bladder pathophysiology. The AC concentration was set at the 50% inhibitory concentration (IC50), as determined by dose-response assays (Fig. S1S). Immunofluorescence images of the 5637 cells demonstrated a increased co-localization of TRPA1 and Na/K-ATPase (membrane markers) in the AC group (Fig. 1N). This observation suggests that TRPA1 channel activity is enhanced under these treatment conditions, potentially implicating its role in altered cellular membrane dynamics and signaling.

Of particular relevance to human pathology, we analyzed urine sediment cells obtained from the midstream urine of the clinical OAB subjects, assessing mRNA expression level of *TRPA1*. Our results indicated that *TRPA1* expression was greatly upregulated in the OAB group compared to the Ctrl group (Fig. 1O). Furthermore, we observed a significant positive correlation between *TRPA1* mRNA level and the severity of OABSS, including urge urinary incontinence score, urgency score, and nocturia score (Fig. 1P). Collectively, the elevated expression of TRPA1 in OAB models and patient samples implicates its role in OAB pathophysiology, thereby highlighting it as a promising therapeutic target.

### Inflammation plays an important role in the pathophysiology of OAB

**Fig. 2:**
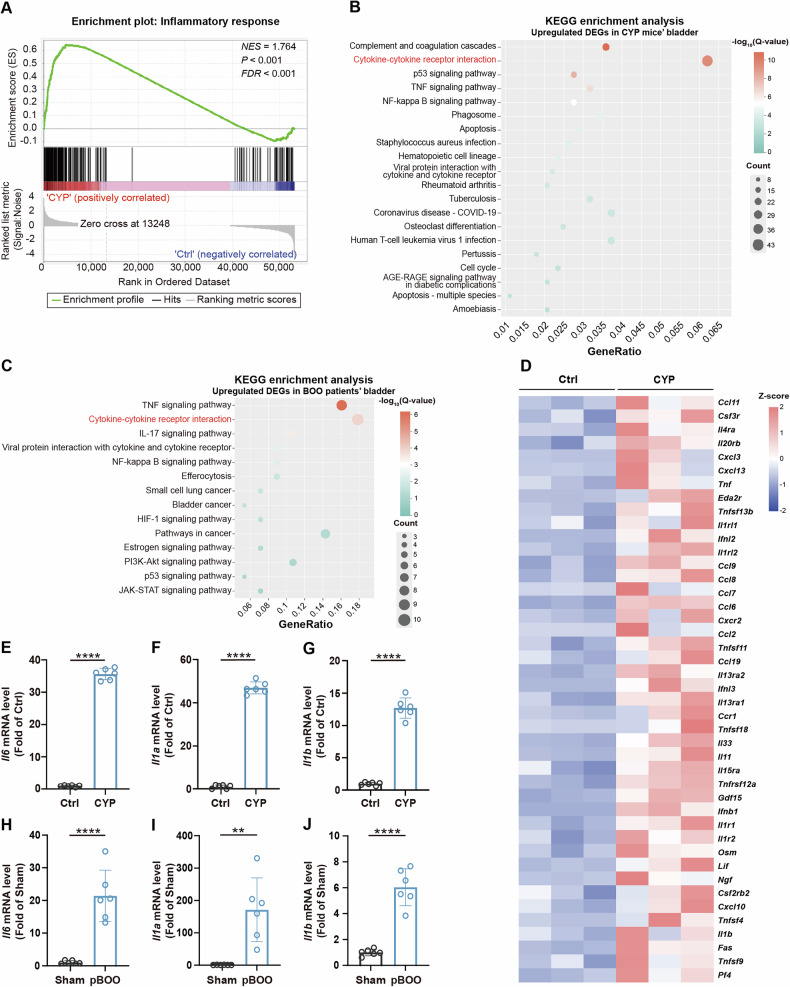
Inflammation is a feature of OAB pathophysiology. **A** GSEA reveals the enrichment of inflammatory response pathways in the bladders of CYP mice. **B** KEGG enrichment analysis highlights the top 20 significantly upregulated signaling pathways identified in the bladders of CYP mice. “Count” represents the number of genes in a gene set that are associated with a specific function or pathway. “GeneRatio” is the ratio of the number of associated genes in the gene set to the total number of genes annotated to that specific function or pathway. **C** A KEGG enrichment analysis of signaling pathways enriched in a human bladder sample (PRJEB11369) indicative of bladder dysfunction is presented. “Count” and “GeneRatio” are defined as previously described. **D** A heatmap illustrates the expression of genes involved in significantly upregulated cytokine-cytokine receptor interaction pathways within the bladders of CYP mice. **E–G** RT-qPCR assays were performed to quantify the mRNA levels of *Il6*, *Il1a*, and *Il1b* in mice’ bladder tissue (n = 6). **H–J** Similar RT-qPCR analyses were conducted to measure the mRNA levels of *Il6*, *Il1a*, and *Il1b* in rats’ bladder samples (*n* = 6). Data are presented as mean ± SD. **P < 0.01 or ****P < 0.0001 using unpaired *t* test.

To explore the gene functions associated with the OAB phenotype, we performed GSEA on the DEGs obtained from the bladder of OAB mice. The result indicates a significant positive enrichment of genes associated with the inflammatory response pathway, with the gene set exhibiting an overall upregulated trend (Fig. 2A). Subsequently, we performed KEGG pathway enrichment analysis and functional annotation of the upregulated DEGs from the bladder of OAB mice, correlating these genes with known metabolic pathways and biological functions. Notably, our findings revealed significant enrichment of inflammation-related pathways, particularly the cytokine-cytokine receptor interaction pathway (Fig. 2B). Additionally, we performed KEGG enrichment analysis on the sequencing data of human bladder dysfunction samples from the European Nucleotide Archive (PRJEB11369), this analysis similarly demonstrated significant enrichment of inflammation-related pathways, including the cytokine-cytokine receptor interaction pathway (Fig. 2C). Heat maps illustrated that inflammatory genes associated with this pathway, such as *Il1b* and *Tnf*, were markedly upregulated in the bladder of CYP mice (Fig. 2D). Consistent with these findings, both CYP mice and pBOO rats exhibited dramatic increases in inflammation, as evidenced by elevated mRNA levels of *Il6*, *Il1a*, and *Il1b* (Fig. 2E-J). These results collectively suggest that inflammatory genes and pathways related to the inflammatory response are considerably upregulated in the OAB models.

### TRPA1 mediates inflammation and bladder dysfunction in OAB mice

**Fig. 3:**
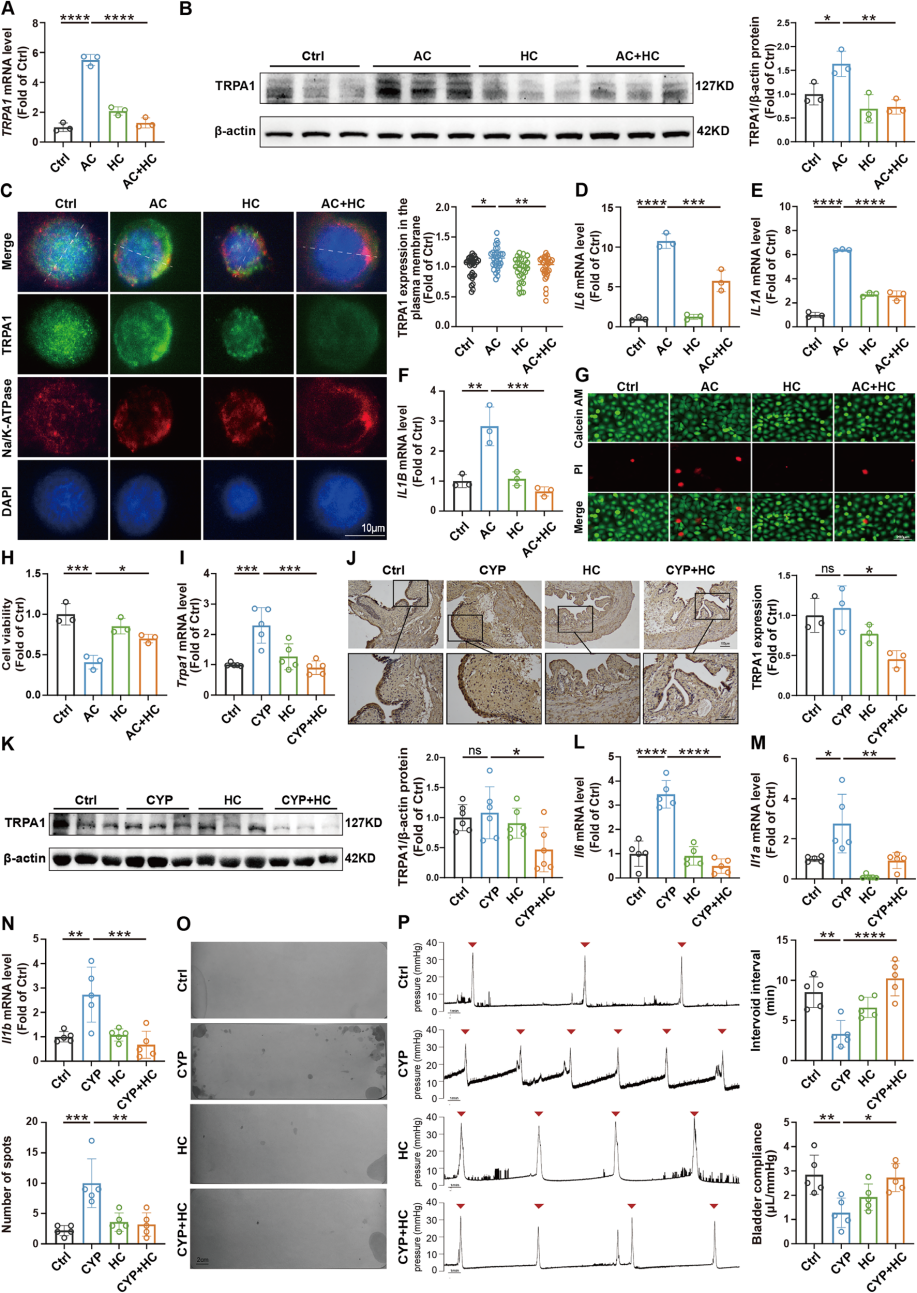
TRPA1 drives OAB-related inflammation and bladder dysfunction. **A** RT-qPCR analysis was conducted to quantify the mRNA levels of *TRPA1* in 5637 cells (*n* = 3). **B** Western blot analysis assessed TRPA1 protein expression in 5637 cells (*n* = 3). **(C)** Immunofluorescence co-localization and line scan were performed to evaluate TRPA1 expression at the plasma membrane of cells, with red representing Na/K-ATPase staining, green indicating TRPA1 staining, and blue denoting nuclear staining (*n* = 30). White lines indicate the locations used for line scan analysis. **D–F** RT-qPCR assays were carried out to measure the mRNA levels of *IL6*, *IL1A*, and *IL1B* in cells (*n* = 3). **G** Propidium iodide (PI) staining was utilized to assess the viability of cells. **H** LDH assay was performed to further evaluate cell viability. **I** RT-qPCR was conducted to quantify the mRNA levels of *Trpa1* in mice’ bladder tissue (*n* = 5). **J** IHC staining was performed to detect TRPA1 expression in mouse bladder. **K** Western blot analysis quantified the protein expression levels of TRPA1 in mice’ bladder tissue (*n* = 6). **L–N** RT-qPCR was conducted to quantify the mRNA levels of *Il6*, *Il1a*, *Il1b* in mice’ bladder tissue (*n* = 5). **O** Viod spot assays were conducted to assess urination behavior in mice, specifically counting urine spots (*n* = 5). **P** Urodynamic test was utilized to evaluate intervoid interval, and bladder compliance in mice (*n* = 5). Data are presented as mean ± SD. *P < 0.05, **P < 0.01, ***P < 0.001, ****P < 0.0001 or ns using one-way ANOVA, followed by Tukey’s post hoc test.

To further elucidate the role of TRPA1 in bladder dysfunction, we employed HC-030031, a selective and specific TRPA1 antagonist, to block TRPA1 channel activity and function following CYP or AC induction. In vitro, HC-030031 significantly attenuated AC-induced upregulation of TRPA1 expression in 5637 cells (Fig. 3A-C), and markedly downregulated inflammation, as indicated by reduced mRNA levels of *IL6*, *IL1A*, and *IL1B* (Fig. 3D-F). Additionally, HC-030031 notably rescued the AC-reduced reduction in cell viability (Fig. 3G, H). Similarly, in vivo, HC-030031 markedly reduced TRPA1 mRNA and protein expression in the bladder tissues of CYP-treated mice (Fig. 3I-K) and ameliorated inflammation (Fig. 3L-N).

In CYP-induced OAB mice, functionally, HC-030031 treatment decreased the number of void spots without altering total urine volume, indicating improved voiding control (Fig. 3O and Fig. S2A). Urodynamic test further revealed that HC-030031 improved the intervoid intervals, bladder compliance, resting pressure, and threshold pressure in CYP mice, while pressure amplitude and peak pressure remained unaffected (Fig. 3P and Fig. S2B-E). Additionally, pharmacological inhibition of TRPA1 reduced bladder edema and lowered the bladder weight to body weight ratio, although the reduction in bladder weight did not achieve statistical significance (Fig. S2F-H). Overall, these findings indicate that TRPA1 plays a critical role in mediating inflammation and regulating bladder function in the context of OAB.

### TRPA1 enhances NLRP3 inflammasome activation and drives pyroptosis in OAB

Prior investigations have underscored the pivotal role of the NLRP3 inflammasome in orchestrating inflammatory processes in models of bladder outlet obstruction and diabetic bladder dysfunction [26, 27]. Upon activation, the NLRP3 inflammasome promotes Caspase-1 activation, which subsequently cleaves GSDMD to form plasma membrane pores, and processes pro-IL-1β and pro-IL-18 into their bioactive forms, thereby facilitating the release of pro-inflammatory cytokines and inducing pyroptosis [28]. In this study, our prior transcriptomic analyses revealed a significant upregulation of *Nlrp3* mRNA levels in the bladders of CYP mice (Fig. 1F). This observation suggests that the NLRP3 inflammasome may serve as a pivotal regulator in bladder dysfunction by triggering inflammation through pyroptosis. Accordingly, we further explored whether TRPA1 modulates inflammation in OAB by regulating NLRP3 inflammasome activity and associated pyroptotic process.

**Fig. 4:**
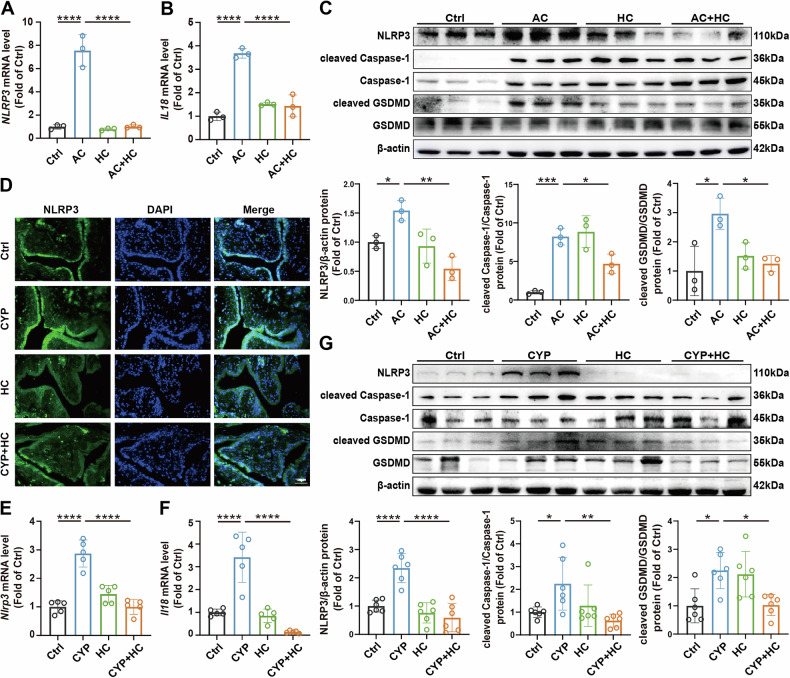
TRPA1 promotes NLRP3 inflammasome activation and pyroptosis in OAB. **A**, **B** RT-qPCR assays were conducted to measure the mRNA levels of *NLRP3* and *IL18* in 5637 cells (*n* = 3). **C** Western blot analyses were performed to detect the protein expression levels of NLRP3, cleaved Caspase-1, and cleaved GSDMD in cells (*n* = 3). **D** IF staining was utilized to assess the localization and expression of NLRP3 in mice’ bladder tissue. **E**, **F** RT-qPCR was performed to quantify the mRNA levels of *Nlrp3* and *Il18* in mice’ bladder samples (n = 5). **G** Western blot analyses were conducted to evaluate the protein expression levels of NLRP3, cleaved Caspase-1, and cleaved GSDMD in mice bladders (*n* = 6). Data are presented as mean ± SD. *P < 0.05, **P < 0.01, ***P < 0.001 or ****P < 0.0001 using one-way ANOVA, followed by Tukey’s post hoc test.

We found that treatment with HC-030031 led to a reduction in the mRNA levels of *NLRP3*, *IL1B*, and *IL18* induced by AC in vitro (Fig. 3F and Fig. 4A, B). Moreover, the increase of protein level of NLRP3, cleaved Caspase-1, and cleaved GSDMD induced by AC, was also significantly diminished by HC-030031 (Fig. 4C). To corroborate the observations in vitro, we subsequently evaluated the expression of relevant factors in CYP mice. IF staining revealed that NLRP3 was predominantly expressed in the bladder urothelium, with its level significantly upregulated in CYP mice, whereas its expression was reduced following HC-030031 treatment (Fig. 4D). Additionally, in vivo experiments demonstrated that HC-030031 effectively inhibited the elevated levels of *Il1b*, *Il18*, and NLRP3, cleaved Caspase-1, cleaved GSDMD in the bladder of CYP mice (Fig. 3N and Fig. 4E-G). These data collectively suggest that TRPA1 enhances NLRP3 inflammasome activity and promotes pyroptosis, further implicating its role in bladder inflammation and dysfunction. The ability of HC-030031 to suppress these pathways underscores the therapeutic potential of targeting TRPA1 to mitigate inflammasome-driven pathology in bladder disorders.

### TRPA1 regulates bladder dysfunction in OAB via NLRP3 inflammasome-dependent mechanism

**Fig. 5:**
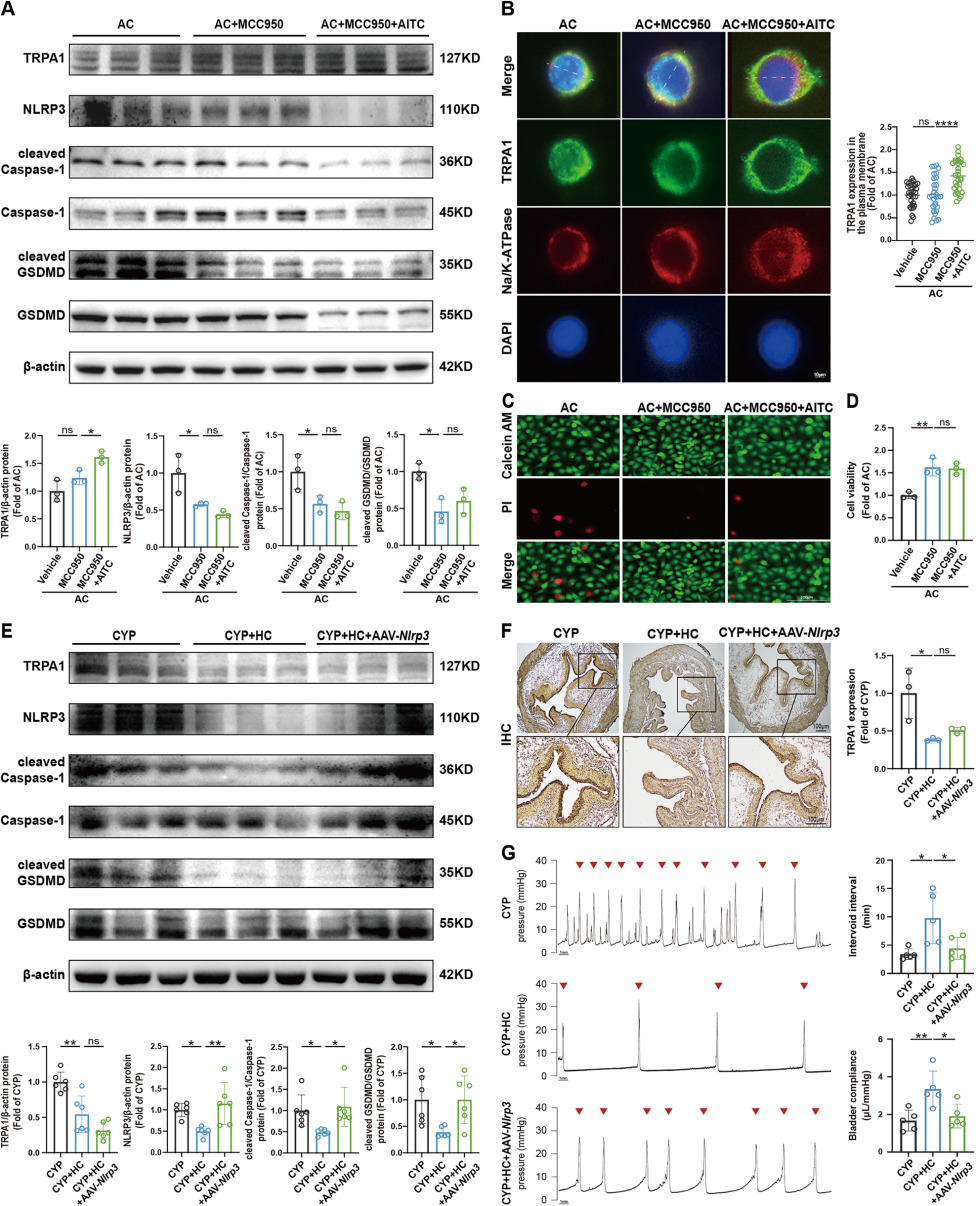
TRPA1 impairs bladder function via the NLRP3 inflammasome. **A** Western blot analyses were conducted to evaluate the protein expression levels of TRPA1, NLRP3, cleaved Caspase-1, and cleaved GSDMD in 5637 cells (*n* = 3). **B** Immunofluorescence co-localization and line scan revealed TRPA1 expression at the plasma membrane of 5637 cells, with red indicating Na/K-ATPase staining, green representing TRPA1 staining, and blue denoting nuclear staining (*n* = 30). White lines indicate the locations used for line scan analysis. **C** PI staining was used to assess the viability of cells. **D** The LDH assay was performed to further evaluate the viability of cells. **E** Western blot analyses were completed to determine the protein expression levels of TRPA1, NLRP3, cleaved Caspase-1, and cleaved GSDMD in mice bladders (*n* = 6). **F** IHC staining was performed to assess TRPA1 expression in mice bladders. **G** Urodynamic test evaluated intervoid interval, and bladder compliance in mice (*n* = 5). Data are presented as mean ± SD. *P < 0.05, **P < 0.01, ****P < 0.0001 or ns using one-way ANOVA, followed by Tukey’s post hoc test.

To determine whether NLRP3 serves as a downstream effector of TRPA1, we co-treated 5637 cells in vitro with MCC950 (a selective NLRP3 inhibitor) and AITC (a TRPA1 agonist) in the context of AC exposure. Subsequently, we evaluated the expression levels of TRPA1, NLRP3 inflammasome components, pyroptosis markers, and downstream inflammatory mediators. The results indicated that MCC950 potently suppressed the mRNA levels of *NLRP3*, *IL18*, *IL1B*, *IL1A*, and *IL6*, with this inhibition remaining unaltered by AITC (Fig. S3A-E). Notably, MCC950 exerted no influence on *TRPA1* mRNA level, which was augmented by AITC treatment (Fig. S3F). Concordantly, the protein abundances of TRPA1, NLRP3, cleaved caspase-1, and cleaved GSDMD paralleled these mRNA patterns (Fig. 5A). As expected, AITC promoted TRPA1 translocation to the plasma membrane, and MCC950 did not affect this process (Fig. 5B). Moreover, the cytoprotective effects of MCC950 on cell viability remained unchanged in the presence of AITC (Fig. 5C, D). Collectively, these observations indicate that TRPA1-mediated signaling in OAB is intricately intertwined with NLRP3 inflammasome activation and pyroptosis, underscoring a multifaceted interplay among these pathways.

To further investigate whether TRPA1 regulates bladder function in CYP mice through modulation of the NLRP3 inflammasome, we administered AAV9 specifically designed to induce overexpression of *Nlrp3* in the bladder. We then assessed the bladder function of these mice. The results demonstrated a significant upregulation of NLRP3 in the bladders of mice in the CYP + HC + AAV9-*Nlrp3* group compared to those in the CYP + HC group (Fig. 5E and Fig. S3G), confirming the successful overexpression of *Nlrp3*. Notably, the inhibitory effects of HC-030031 on NLRP3 inflammasome activity, pyroptosis process and inflammation were markedly abrogated by AAV-*Nlrp3*, as evidenced by significant differences in the expression levels of NLRP3, cleaved Caspase-1, cleaved GSDMD, and *Il18*, *Il1b*, *Il1a* and *Il6* between the CYP + HC group and the CYP + HC + AAV-*Nlrp3* group (Fig. 5E and Fig. S3H-K). Importantly, *Nlrp3* overexpression did not alter TRPA1 expression (Fig. 5E, F and Fig. S3L). Furthermore, the structural improvements in bladder tissue, such as the reduction of lamina propria edema induced by HC-030031, were also negated by AAV-*Nlrp3* (Fig. S3M).

Urodynamic analysis revealed that HC-030031 significantly enhanced bladder function in CYP mice, as demonstrated by prolonged intervoid interval, improved bladder compliance, and reduced resting and threshold pressures, without affecting pressure amplitude or peak pressure (Fig. 5G and Fig. S3N-Q). In contrast, the CYP + HC + AAV9-*Nlrp3* group exhibited urodynamic parameters comparable to those of the CYP group (Fig. 5G and Fig. S3N, O), indicating that *Nlrp3* overexpression negated the therapeutic benefits of TRPA1 inhibition. Collectively, these results demonstrate that TRPA1 modulates bladder dysfunction in OAB through an NLRP3 inflammasome-dependent pathway, highlighting the critical role of this molecular axis in regulating inflammation and bladder function.

### TRPA1 promotes NLRP3 expression via the transcription factors MAZ and SMAD3

**Fig. 6:**
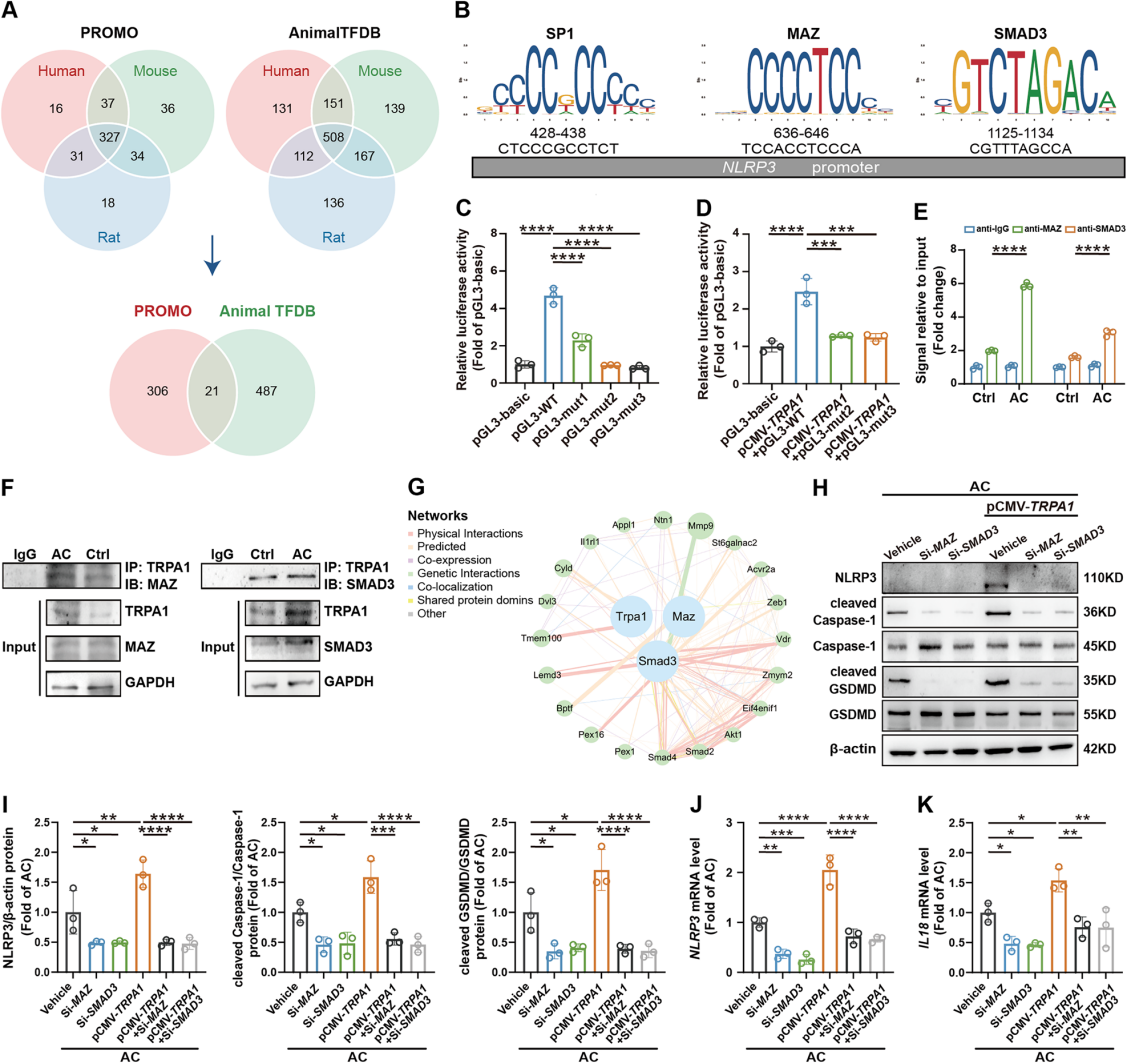
MAZ and SMAD3 mediate TRPA1-induced NLRP3 upregulation. **A** Venn analysis identifying transcription factors associated with *NLRP3*. **B** Prediction of binding sites for SP1, MAZ, and SMAD3 in the *NLRP3* promoter. **C**, **D** Dual luciferase reporter assays were conducted to evaluate the transcriptional activity of *NLRP3* (*n* = 3). **E** ChIP assay was performed to quantify the binding of transcription factors to the *NLRP3* promoter in 5637 cells (*n* = 3). **F** Co-IP was employed to detect the binding levels of TRPA1 to transcription factors in 5637 cells. **G** GeneMANIA was utilized to predict interactions between TRPA1 and MAZ, SMAD3. Each node represents a gene, and its size corresponds to the gene’s significance. The colors of the edges between nodes denote the type of functional interaction, as detailed in the figure, while the thickness of the edges indicates the strength of the association. **H**, **I** Western blot analysis was conducted to assess the protein expression levels of NLRP3, cleaved Caspase-1, and cleaved GSDMD in cells (*n* = 3). **J**, **K** RT-qPCR analyses were carried out to measure the mRNA levels of *NLRP3*, *IL18* in cells (*n* = 3). Data are presented as mean ± SD. *P < 0.05, **P < 0.01, ***P < 0.001 or ****P < 0.0001 using one-way ANOVA, followed by Tukey’s post hoc test.

Given that TRPA1 can regulate the expression of NLRP3 at both transcriptional and translational levels in vitro and in vivo, we postulated that TRPA1 initially modulates *NLRP3* transcription, which subsequently influences the translational expression of the NLRP3 protein. To investigate the specific mechanisms underlying TRPA1’s regulation of *NLRP3* transcription, we conducted a comprehensive screening of transcription factors potentially involved in regulating *NLRP3* in human, mouse and rat using the PROMO database and the AnimalTFDB database. This approach yielded a total of 21 transcription factors following the identification of common factors across species (Fig. 6A). Upon reviewing pertinent literature [29–31], we identified three transcription factors—SP1, MAZ and SMAD3—which may interact with TRPA1 and play roles in *NLRP3* regulation. Potential binding sites of these transcription factors within the *NLRP3* promoter region were predicted using the JASPAR database (Fig. 6B). Subsequently, we created mutant constructs of the *NLRP3* promoter to elucidate the functional significance of these predicted interactions. To evaluate the impact of *NLRP3* promoter mutations on its transcriptional activity, a dual luciferase reporter assay was conducted. The results indicated that mutations in the binding sites of SP1, MAZ, and SMAD3 decreased the transcriptional activity of *NLRP3* compared to the unmutated wild-type (WT). Notably, the mutations of MAZ and SMAD3 binding sites led to a more marked reduction in *NLRP3* transcriptional activity (Fig. 6C). Subsequently, we transfected a *TRPA1* overexpression plasmid in the context of these two mutant constructs. Despite overexpressing *TRPA1*, the transcriptional activity of *NLRP3* did not recover from the declines caused by the mutations of MAZ and SMAD3 binding sites (Fig. 6D). Moreover, in vitro studies demonstrated that AC treatment enhanced the binding of both MAZ and SMAD3 to the *NLRP3* promoter region (Fig. 6E). Furthermore, we found that AC treatment also amplified the physical interaction between TRPA1 and these two transcription factors (Fig. 6F). The potential for the functional interplay among TRPA1, MAZ, and SMAD3 was further supported by predictions from the online analysis tool GeneMANIA (https://genemania.org) (Fig. 6G). Furthermore, silencing *MAZ* or *SMAD3* significantly reduced the expression levels of NLRP3, cleaved Caspase-1, cleaved GSDMD, and *IL18*, overexpression of *TRPA1* could not reverse the downregulation of these factors (Fig. 6H-K and Fig. S4A-C). Collectively, these data support that TRPA1’s regulatory effects on the NLRP3 inflammasome and pyroptosis are contingent upon MAZ and SMAD3.

## Discussion

Although the presence of TRPA1 in the bladder and its role in urinary pathophysiology and voiding function have been documented, the precise mechanisms governing its activity and associated inflammatory processes remain incompletely understood. This study elucidates the progressive upregulation of TRPA1 expression in conjunction with the severity and duration of OAB, positioning TRPA1 as a potential biomarker for assessing OAB severity. Mechanistic investigations reveal that TRPA1 modulates inflammation by enhancing the NLRP3 inflammasome activity and pyroptosis process in OAB, suggesting a critical interplay between TRPA1 and NLRP3. Further, our findings indicate that TRPA1 regulates NLRP3 through the action of transcription factors MAZ and SMAD3. Notably, the TRPA1 inhibitor HC-030031 not only attenuates pyroptotic activity and inflammatory responses but also fosters urothelial regeneration and restores bladder function. These outcomes highlight the therapeutic promise of targeting TRPA1 in managing urinary dysfunction associated with OAB, thereby opening avenues for innovative treatment strategies in this prevalent condition.

In this investigation, we delineated the pivotal role of TRPA1 in OAB by demonstrating elevated *Trpa1* mRNA levels across diverse OAB models and bladder dysfunction conditions, consistent with prior investigations [32, 33]. Notably, we observed a positive correlation between *TRPA1* expression and the severity of OAB in human subjects. This finding aligns with results from animal models, where TRPA1 protein expression was not significantly upregulated in the CYP mouse model, which represents an initial stage of the disorder, but was markedly upregulated in the pBOO rat model that simulates a more advanced stage of OAB. These observations suggest a gradual increase in TRPA1 expression with the progression and duration of OAB. Furthermore, the upregulation of TRPA1 was associated with urothelial structure and morphological alterations in both the OAB mice and rats, alongside impaired bladder function. Both animal models exhibited higher resting and threshold bladder pressures, indicating heightened sensitivity to bladder filling and a reduced urine volume requirement to initiate urination, thereby reflecting an overactive phenotype. Interestingly, while OAB mice did not develop significant fibrosis, the OAB rats did, reinforcing the link between increased TRPA1 expression and bladder structural abnormalities. These findings may elucidate the clinical observation that patients experience more severe symptoms with a longer duration of OAB [34].

Additionally, recent evidence indicates that TRPA1 is localized within mobile intracellular vesicles and traffics to the plasma membrane upon activation [22–25]. In our study, we also noted an elevation in the TRPA1 protein level at the plasma membrane, suggesting a significant enhancement of TRPA1 activity in the OAB models. Moreover, the observed discrepancy between *Trpa1* mRNA level and TRPA1 protein expression may arise from post-transcriptional regulatory mechanisms or protein degradation pathways, which were not explored in this study. Nonetheless, the heightened TRPA1 expression underscores its indispensable role in OAB pathophysiology, positioning TRPA1 as a promising therapeutic target for mitigating OAB symptoms.

Extensive evidence has firmly linked urinary symptoms to the pathogenesis of bladder dysfunction, characterized by heightened bladder inflammation [26, 35, 36]. In the present study, we observed significant enrichment of inflammatory signaling pathways in patients with bladder dysfunction, as well as in OAB animal models, consistent with prior investigations [37, 38]. Existing literature underscores the regulatory role of TRPA1 in inflammatory processes [39]. Specifically, TRPA1 activation promotes the release of neuropeptides from neurons and inflammatory mediators from peripheral tissues, whereas its inhibition curtails the production of pro-inflammatory cytokines [40, 41]. Herein, we demonstrated that pharmacological modulation of TRPA1 expression and activity using the selective inhibitor HC-030031 substantially mitigated inflammation in both AC-treated 5637 cells and the CYP mouse model. This amelioration was manifested by pronounced reductions in the levels of pro-inflammatory cytokines. Notably, previous reports have indicated that inflammatory cascades augment TRPA1 membrane translocation and enhance its activation [22]. However, the bidirectional interplay between TRPA1 and inflammation was not examined in this study. This aspect warrants future investigation to fully understand the complex interplay between these two components in the context of bladder dysfunction.

The anti-inflammatory properties of HC-030031 likely confer protection to urothelial cells, thereby preserving bladder homeostasis. Urothelial cells, forming a specialized stratified epithelium, act as a passive barrier lining the bladder wall [42]. Heightened inflammatory signals can induce increased apoptosis of these cells [43], thereby compromising their integrity and functionality, which will affect the release of urothelial mediators and disrupt the micturition reflex in turn [44]. Consistent with observations by Akaihata et al. and Chen et al. [18, 45], we did note marked loss and disruption of the urothelial layer in the bladders of both OAB mice and rats, accompanied by significant bladder dysfunction. The TRPA1 inhibitor markedly enhanced cell viability in AC-treated cells in vitro, reversed the substantial loss and disruption of the urothelial barrier evident in the bladders of OAB mice, culminating in ameliorated urinary urgency and improved bladder compliance, reinforcing the therapeutic promise of TRPA1 modulation in ameliorating bladder pathology and reinstating normal physiological function. Therefore, targeting TRPA1 emerges as a promising strategy for alleviating inflammation and preserving urothelial integrity in disorders characterized by bladder dysfunction. Although HC-030031 is one of the most commonly used TRPA1 blockers, employing *Trpa1* knockout mice may facilitate a more precise evaluation of TRPA1 function in the context of OAB. Future studies utilizing specific gene deletion models will be crucial for elucidating the critical role of TRPA1 in bladder pathophysiology and advancing our understanding of its potential as a therapeutic target.

Another key novel insight from this study is that TRPA1 modulates inflammation and bladder function in OAB by regulating NLRP3 inflammasome activity and pyroptosis. Mechanistically, NLRP3 activation engages the NLRP3-caspase-1-GSDMD-IL-1β/IL-18 axis to elicit pyroptosis, culminating in urothelial cell disruption. Moreover, pyroptosis-derived IL-1β and IL-18 not only exacerbate fibrosis by activating NF-κB signaling pathways which upregulate fibrogenic genes [46], but also sensitize sensory nerve terminals in the bladder wall, increasing neuronal hypersensitivity and thereby driving the bladder to show the signs of overactivity such as urinary urgency and frequency [47]. Consequently, in OAB, urothelial pyroptosis and bladder dysfunction are interconnected through multifaceted mechanisms, including inflammation, epithelial barrier damage, and neuro-sensory hypersensitivity, all of which are governed by the activity of TRPA1 and the NLRP3 inflammasome. These findings illuminate the critical role of this molecular axis in driving bladder pathology and raise intriguing questions about the precise mechanisms by which TRPA1 promotes NLRP3 activation. Elucidating these interactions may uncover novel therapeutic targets for mitigating inflammation and restoring bladder function in OAB and related lower urinary tract disorders.

It is well established that TRP channels regulate diverse transcription factors, including NF-κB, NFAT, STAT3, and HIF-1, which subsequently regulate the NLRP3 inflammasome. This intricate regulatory network suggests that TRP channels play a significant role in mediating inflammatory responses by modulating the activity of these transcription factors [48]. Our results reveal that TRPA1 modulates NLRP3 expression via the transcription factors MAZ and SMAD3. Existing literature highlights MAZ is critical to the development of bladder [49]. Prior studies have also documented associations between NLRP3 activation and SMAD3 [50, 51]. In the present study, we identified that both MAZ and SMAD3 are closely associated with the transcription of *NLRP3* and the production of the NLRP3 inflammasome. Existing literature indicates that TRPA1 may activate the TGF-β signaling pathway to regulate SMAD3 activity [29, 52]. Although the interplay between TRPA1 and MAZ remains largely uncharted, our data suggest that TRPA1 may form complexes with MAZ and SMAD3, thereby promoting *NLRP3* transcription and augmenting inflammasome production through protein-protein interactions. Notably, siRNA-mediated knockdown of *MAZ* and *SMAD3* abrogated TRPA1-driven *NLRP3* transcription, consequently attenuating inflammation and OAB progression. In summary, these findings elucidate the sophisticated interplay among TRPA1, MAZ, and SMAD3 in regulating *NLRP3* transcription and inflammasome activation, highlighting the therapeutic promise of targeting these pathways in inflammatory bladder disorders such as OAB.

## Supplementary information

Supplemental Material

Original Western blots

## Data Availability

All data collected for the study that underpins the conclusions are presented in the main paper and the Supplementary Materials, and data can be shared by the corresponding author upon reasonable request. The RNA-seq data supporting the findings of this study is openly available in National Center for Biotechnology Information (https://www.ncbi.nlm.nih.gov/bioproject/PRJNA1345051/).
