# Supplementary material for: TRPA1 promotes overactive bladder progression by activating the NLRP3 inflammasome and driving pyroptosis
Source: Cell Death Dis. 2026 Feb 16;17(1):226. doi: 10.1038/s41419-026-08426-5 (PMC12921228; doi:10.1038/s41419-026-08426-5)
Supplement: Supplementary file 1 — Supplemental Material [file 41419_2026_8426_MOESM1_ESM.docx]

**Supplementary Material**

**TRPA1 promotes overactive bladder progression by activating the NLRP3 inflammasome and driving pyroptosis**

Yongjuan Rao *et al.*

*Corresponding author. Email: [junlian_gu@sdu.edu.cn;](mailto:junlian_gu@sdu.edu.cn;) [wangkf@sdu.edu.cn](mailto:wangkf@sdu.edu.cn)

This file contains the following:

Figs. S1 to S4

**Figure S1**

**
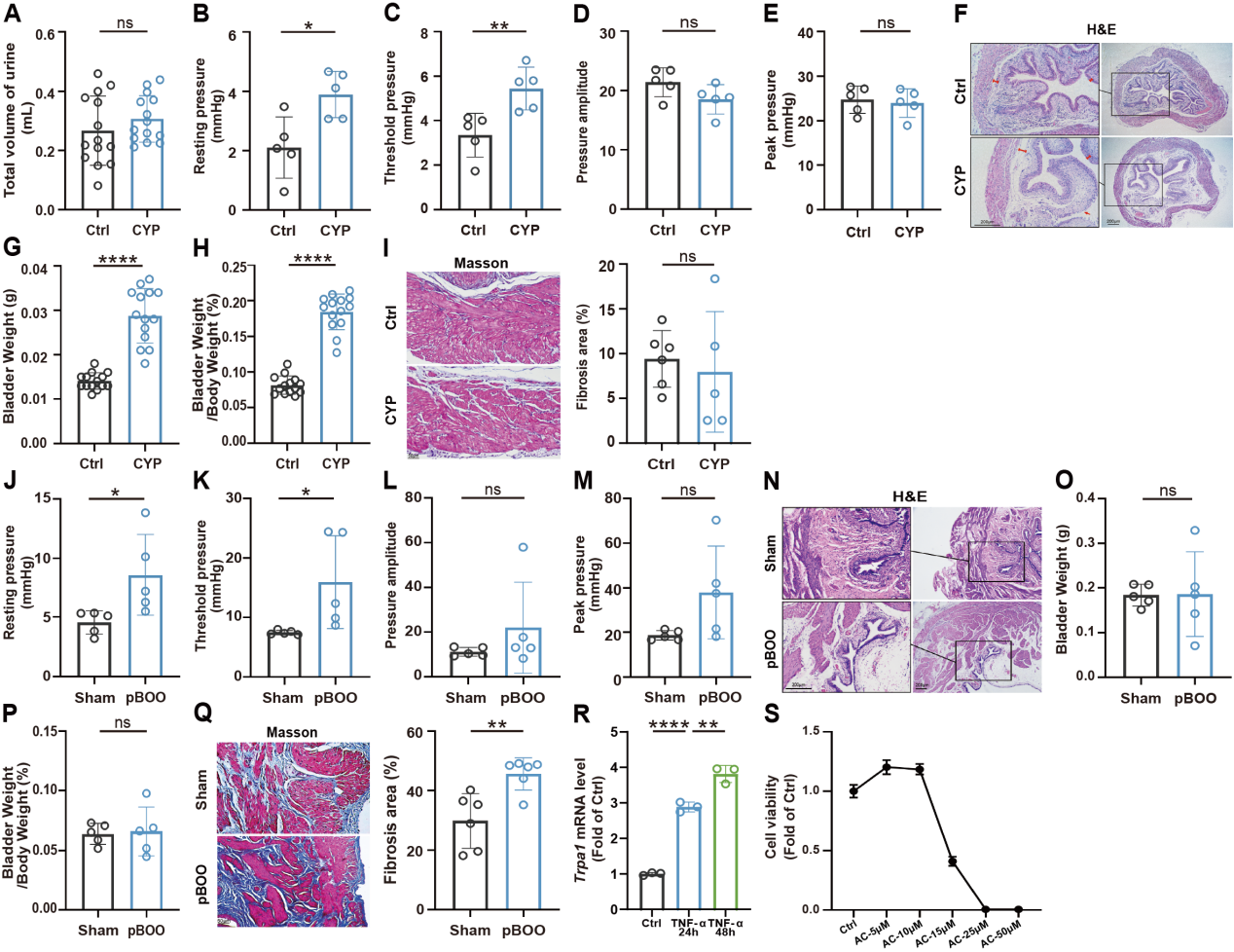
Figure S1: TRPA1 upregulation in OAB bladder. (A)** Void spot assay to assess urination behavior, specifically measuring the total volume of urine in mice (n = 14). **(B-E)** Urodynamic test to monitor key bladder parameters, including resting pressure, threshold pressure, pressure amplitude and peak pressure in mice (n = 5). **(F)** H&E staining to detect structural alterations in the mouse bladder, where the red line segment demonstrates the thickness of the uroepithelium and lamina propria in mice in the CTRL and CYP groups, and the red arrows point to the absence and destruction of the uroepithelial cell layer. **(G, H)** Bladder weight and the ratio of bladder to body weight in mice (n = 14). **(I)** Masson's trichrome staining to detect the level of fibrosis in the bladder of mice (n = 5-6). **(J-M)** Urodynamic test to detect resting pressure, threshold pressure, pressure amplitude and peak pressure in rats (n = 5). **(N)** H&E staining to detect structural alterations in the rats’ bladder tissue. **(O, P)** Bladder weight and the ratio of bladder to body weight in rats (n = 5). **(Q)** Masson's trichrome staining to detect the level of fibrosis in the bladder of rats (n = 6). **(R)** Transcript levels of *Trpa1* in bladder smooth muscle cells in the TNF-α-induced mimicry of in vitro bladder dysfunction dataset (PRJEB24781). **(S)** Cell viability of 5637 cells by CCK-8 assay (n = 3). Data are presented as mean ± SD. **P* < 0.05, ***P* < 0.01, *****P* < 0.0001 or ns using unpaired *t* test.

**Figure S2**

**
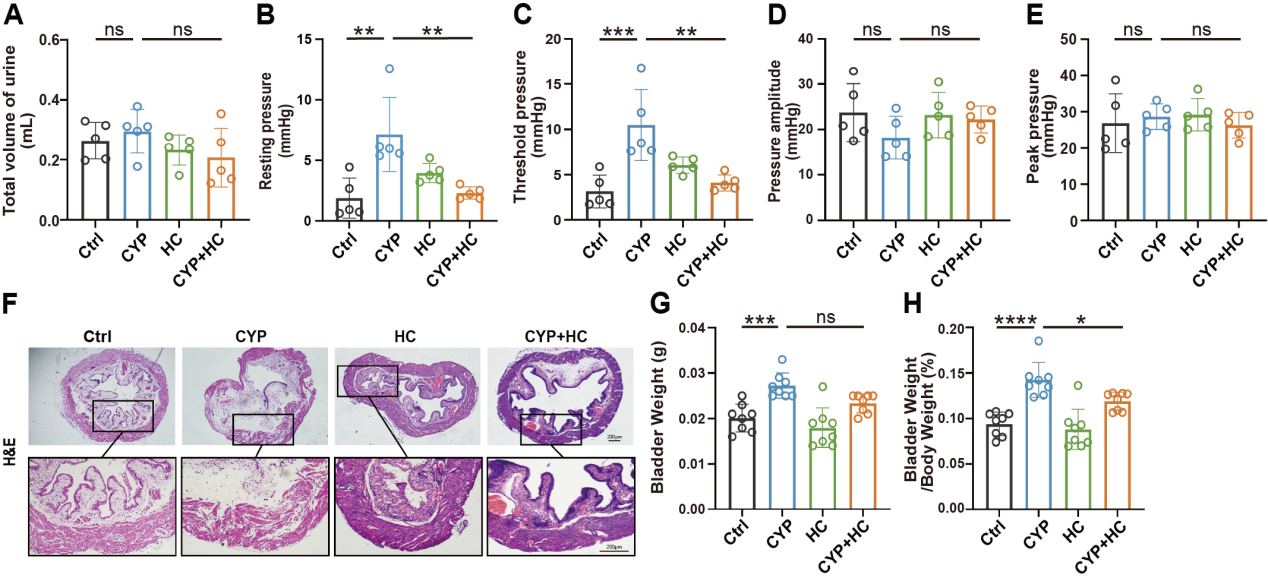
Figure S2. TRPA1 mediates inflammation and bladder dysfunction. (A)** Void spot assay to to assess urination behavior, specifically measuring the total volume of urine in mice (n = 5). **(B-E)** Urodynamic test to monitor key bladder parameters, including resting pressure, threshold pressure, pressure amplitude and peak pressure in mice (n = 5). **(F)** H&E staining to detect structural alterations in the mice bladder. **(G, H)** Bladder weight and the ratio of bladder to body weight in mice (n = 8). Data are presented as mean ± SD. **p* < 0.05, ***p* < 0.01, ****p* < 0.001, *****P* < 0.0001 or ns using one-way ANOVA, followed by Tukey’s post hoc test.

**Figure S3**

**
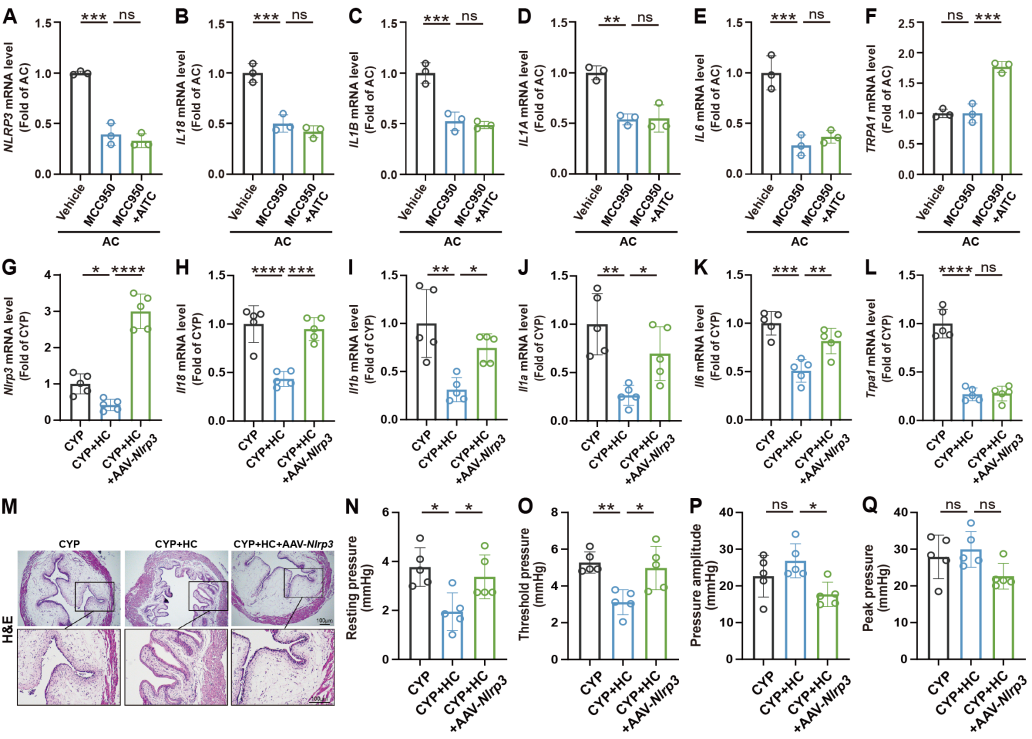
**

**Figure S3. TRPA1 regulates bladder function via the NLRP3 inflammasome. (A-F)** RT-qPCR assays were performed to detect the mRNA levels of *NLRP3*, *IL18*, *IL1B*, *IL1A*, *IL6*, and *TRPA1* in 5637 cells (n = 3). **(G-L)** RT-qPCR was conducted to measure the mRNA levels of *Nlrp3*, *Il18*, *Il1b*, *Il1a*, *Il6* in mice’ bladder tissue (n = 5). **(M)** H&E staining to detect structural alterations in the mice bladder. (**N-Q**) Urodynamic test to monitor key bladder parameters, including resting pressure, threshold pressure, pressure amplitude and peak pressure in mice (n = 5). Data are presented as mean ± SD. **p* < 0.05, ***p* < 0.01, ****p* < 0.001, *****P* < 0.0001 or ns using one-way ANOVA, followed by Tukey’s post hoc test.

**Figure S4**

**
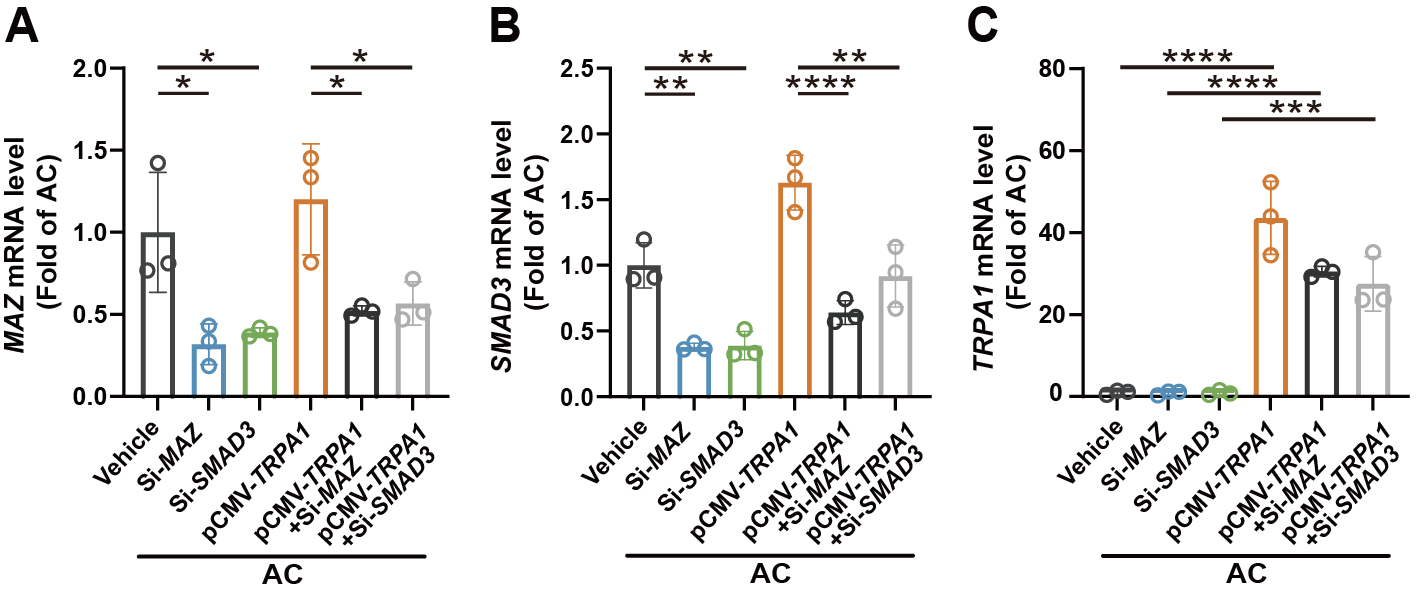
**

**Figure S4. TRPA1 upregulates NLRP3 through MAZ and SMAD3. (A-C)** RT-qPCR assays were performed to detect the mRNA levels of *MAZ*, *SMAD3* and *TRPA1* in 5637 cells (n = 3). Data are presented as mean ± SD. **P* < 0.05, ***P* < 0.01, ****P* < 0.001 or *****P* < 0.0001 using one-way ANOVA, followed by Tukey’s post hoc test.
