# Supplementary material for: TRPA1 promotes overactive bladder progression by activating the NLRP3 inflammasome and driving pyroptosis
Source: Cell Death Dis. 2026 Feb 16;17(1):226. doi: 10.1038/s41419-026-08426-5 (PMC12921228; doi:10.1038/s41419-026-08426-5)

Full unedited gel for Figure 1J

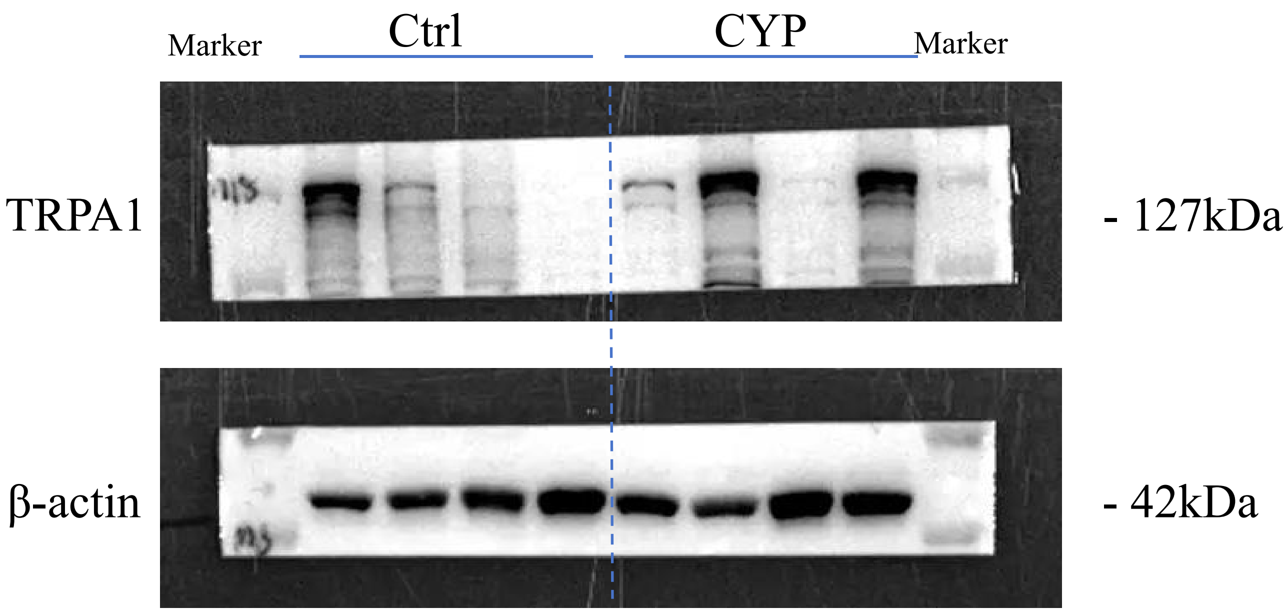

Figure 1J copy

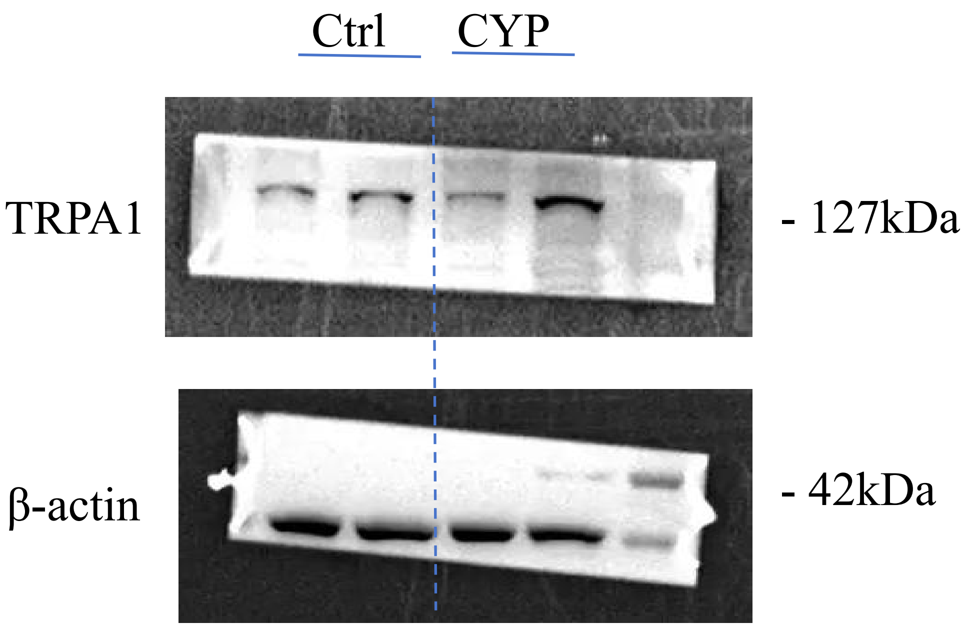

Full unedited gel for Figure 1L

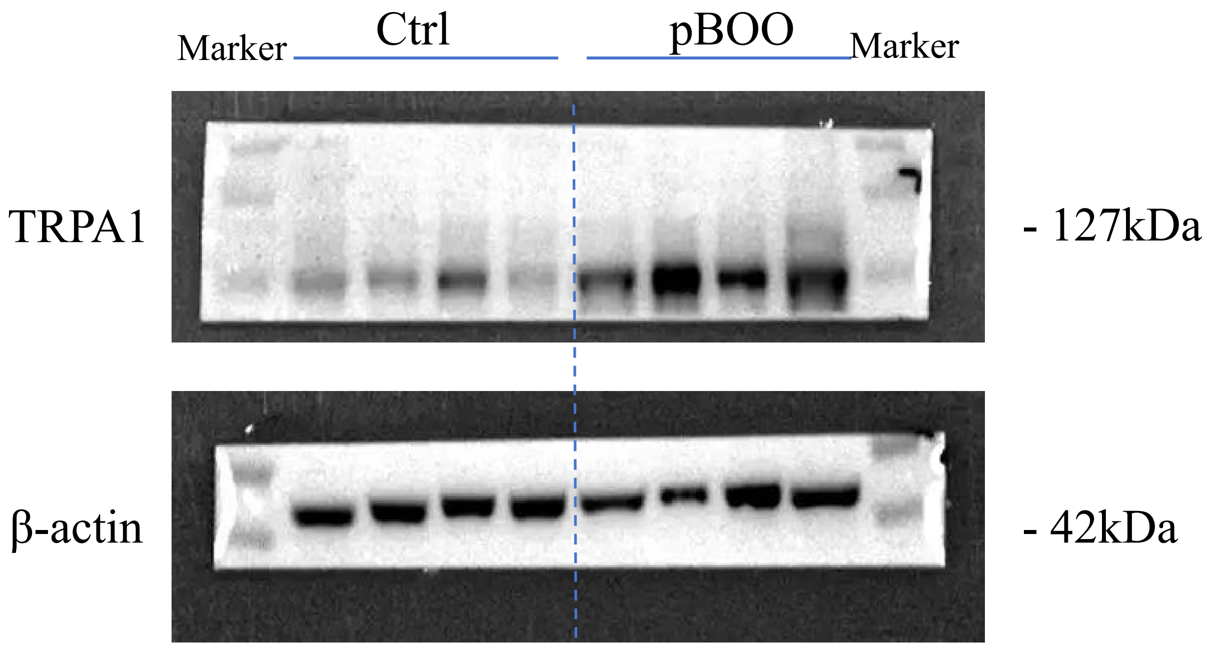

Figure 1L copy

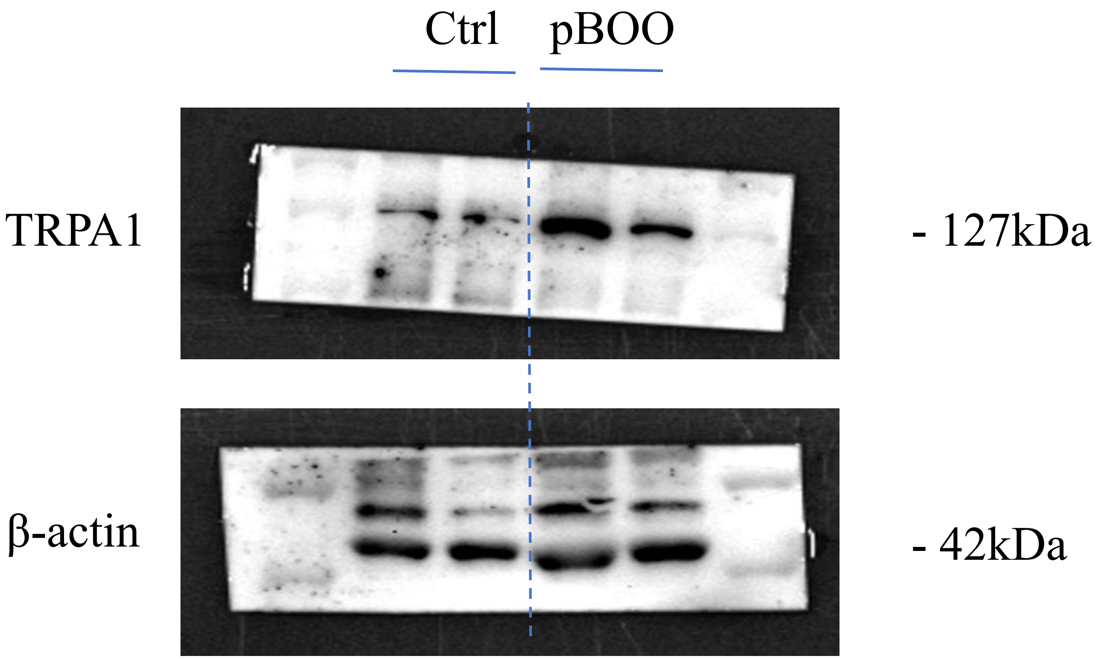

Full unedited gel for Figure 3B

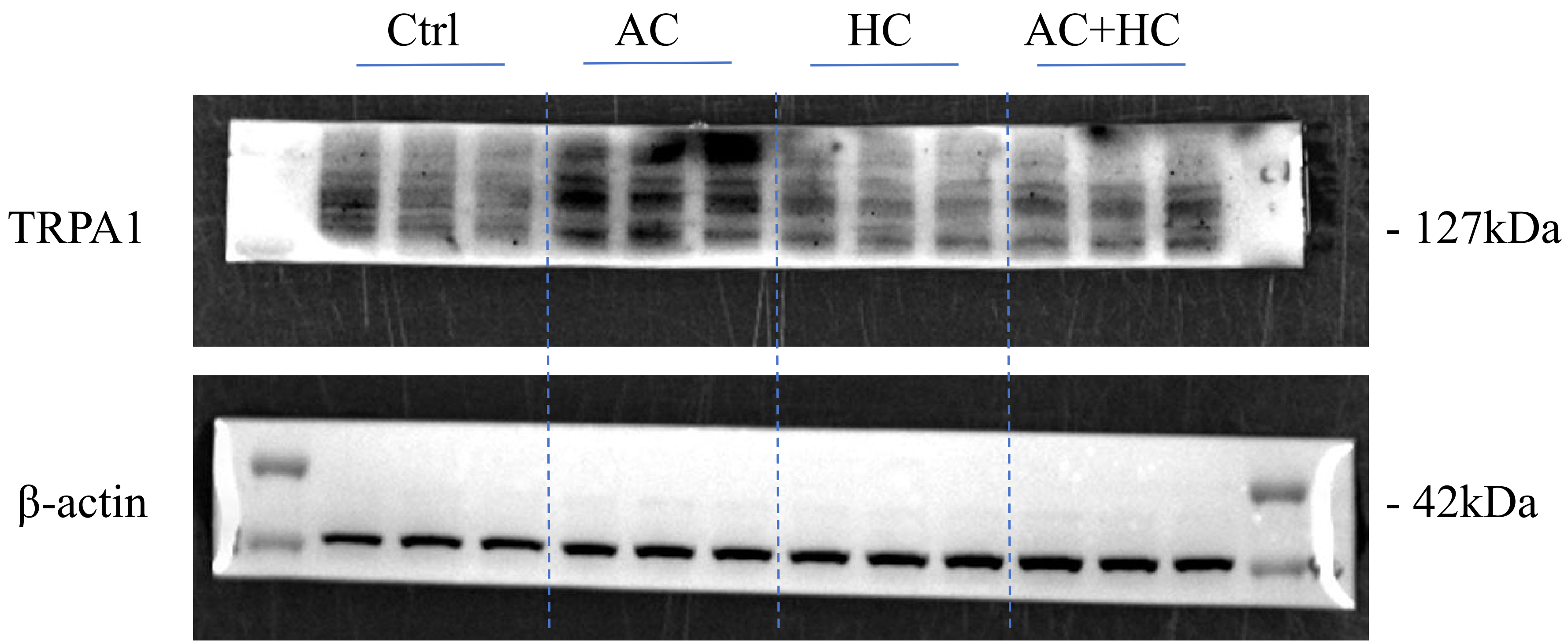

Full unedited gel for Figure 3K

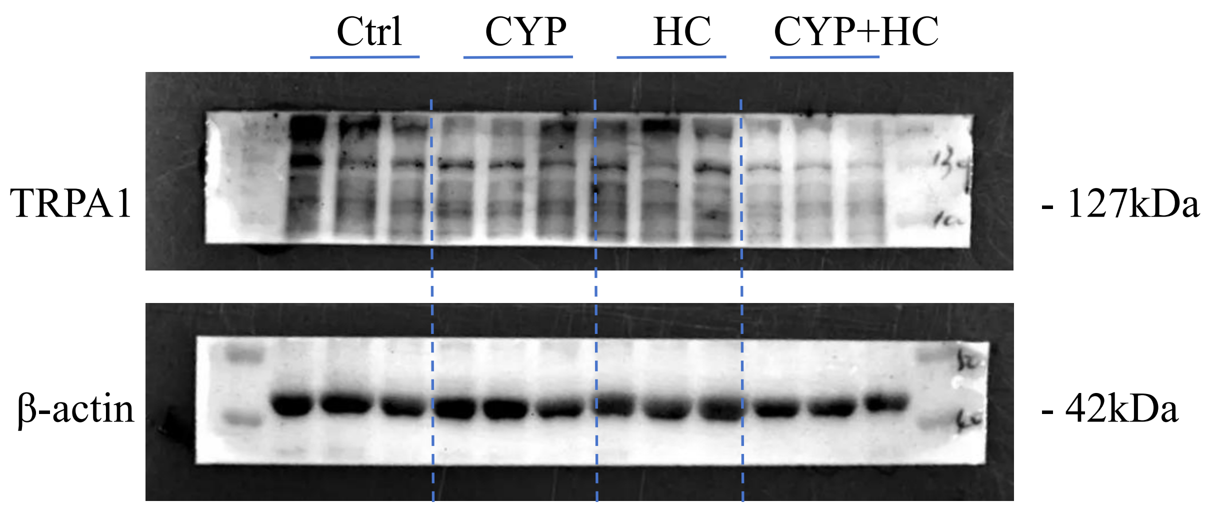

Figure 3K copy

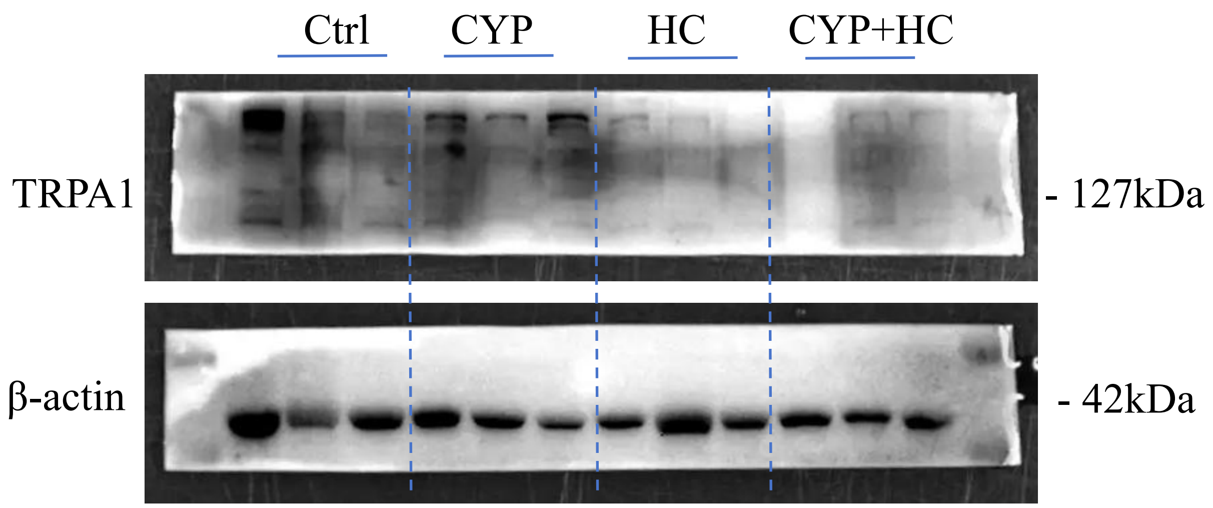

Full unedited gel for Figure 4C

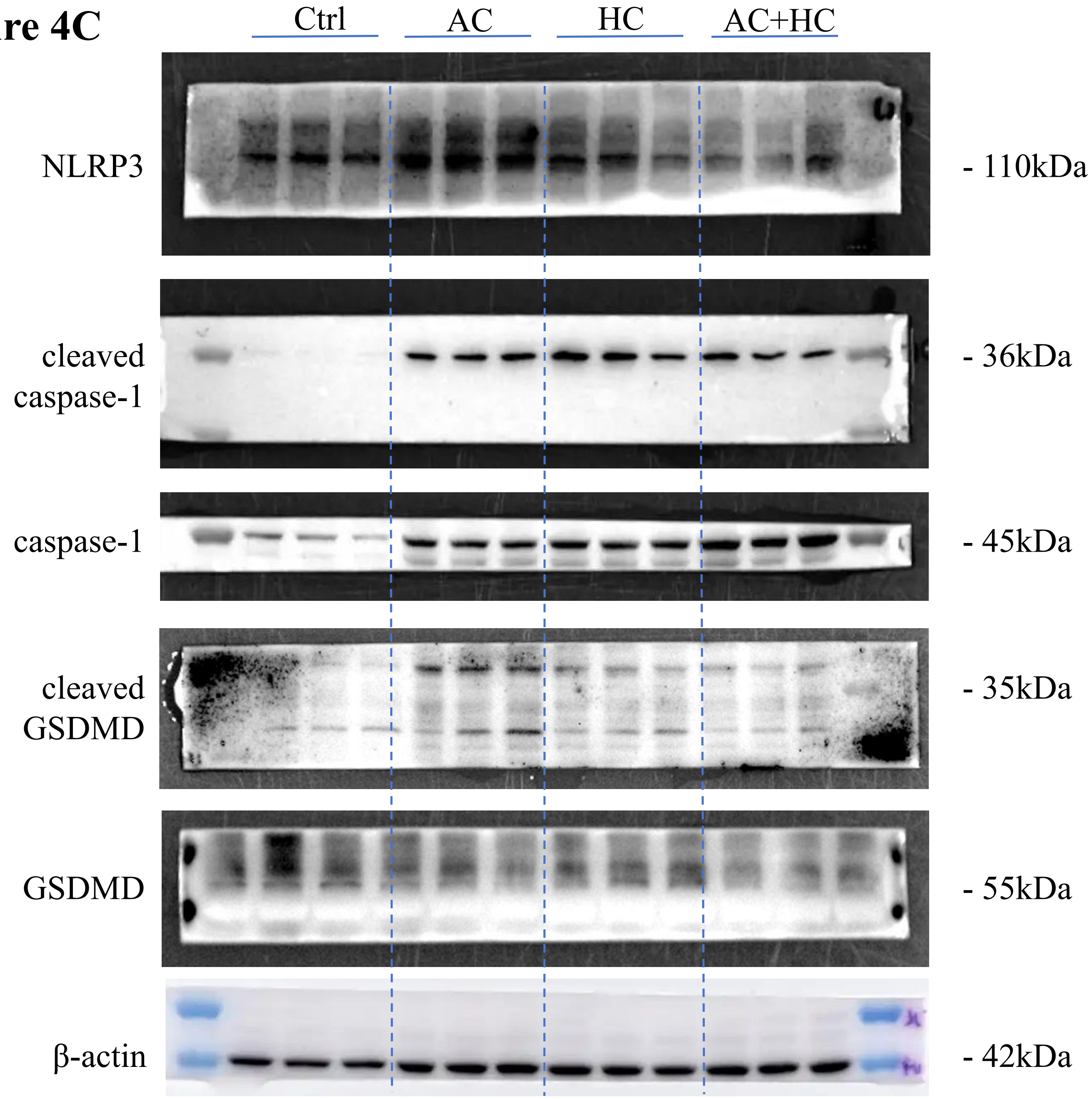

Full unedited gel for Figure 4G

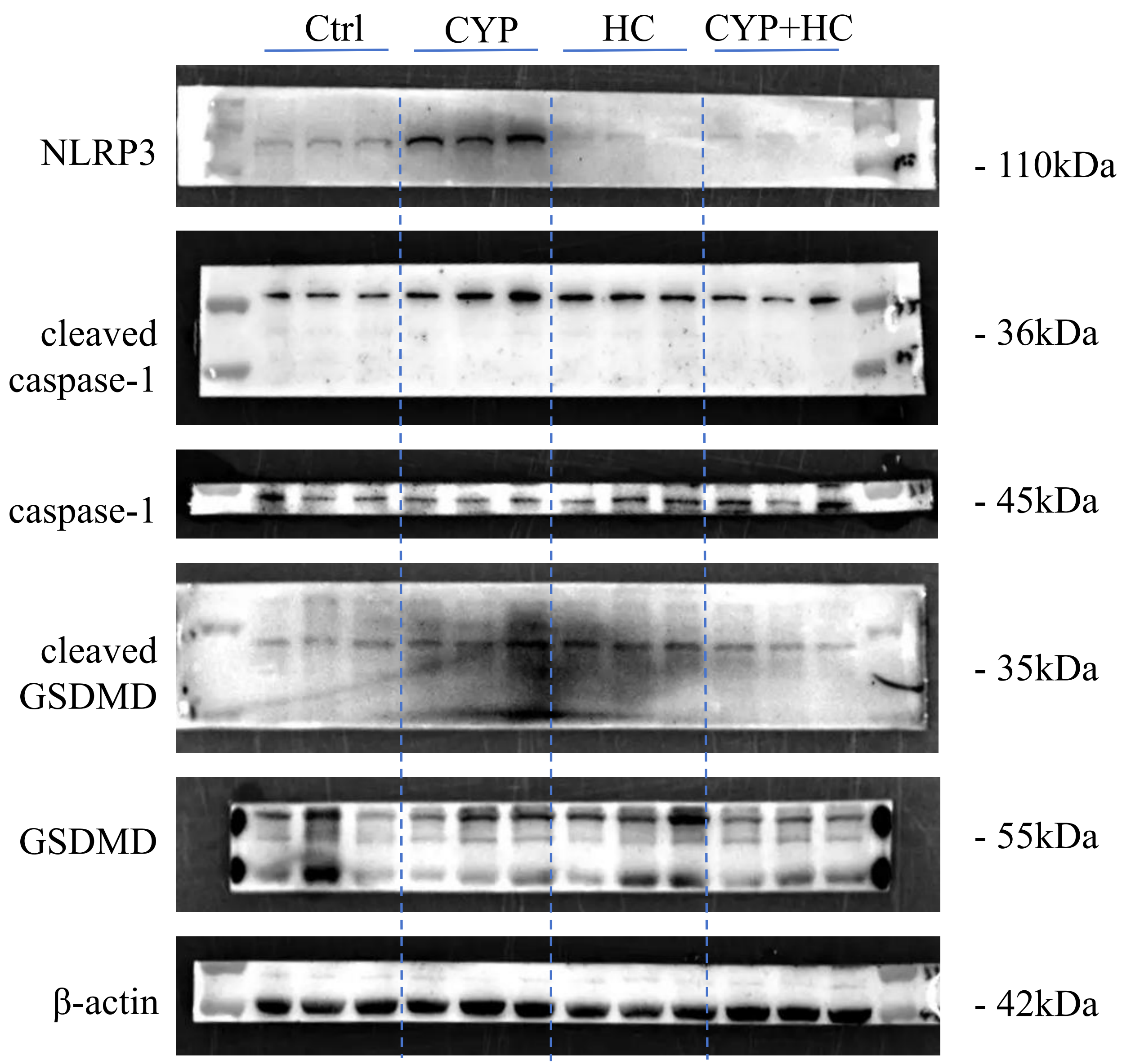

Figure 4G copy

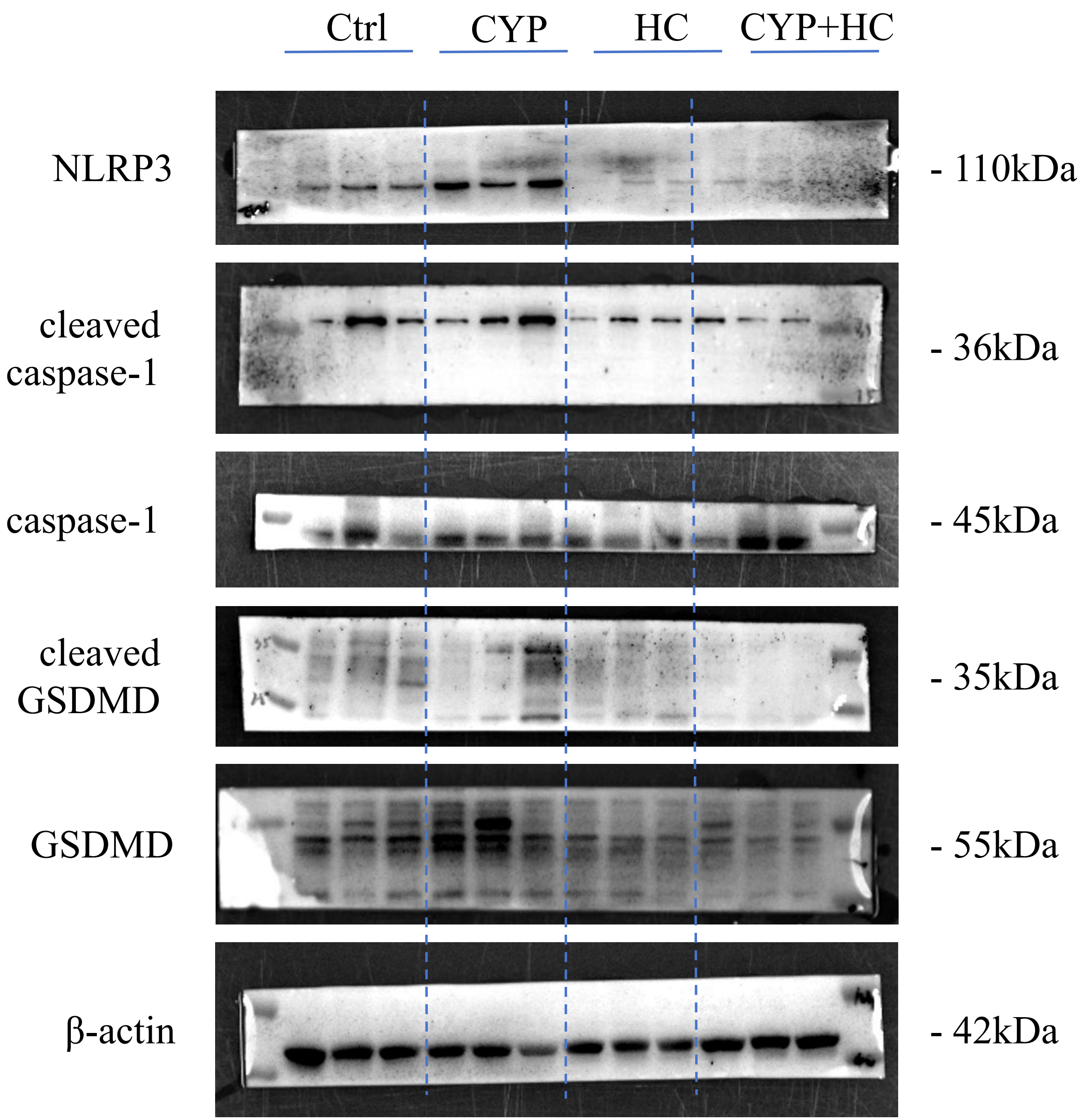

Full unedited gel for Figure 5A

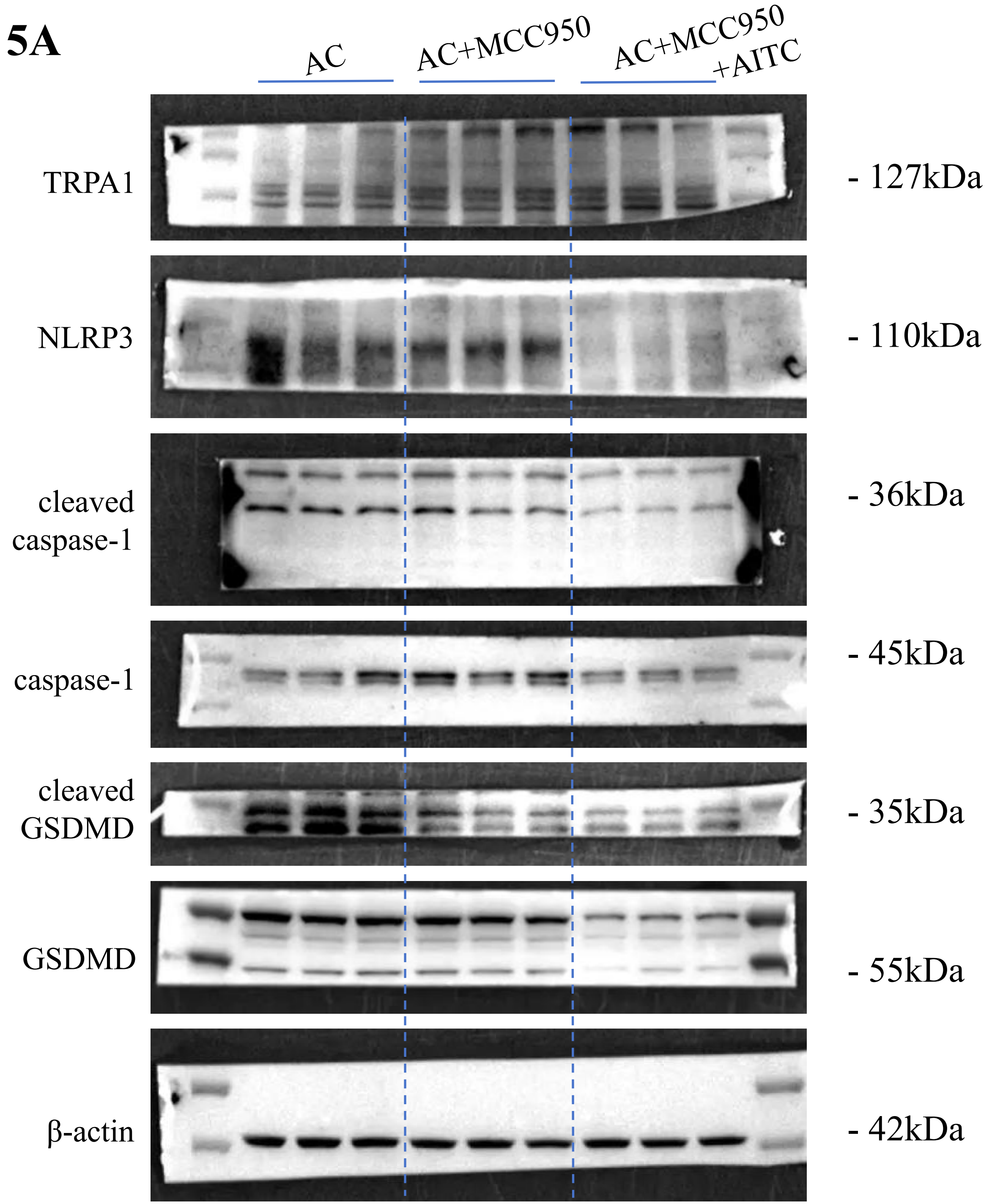

Full unedited gel for Figure 5E

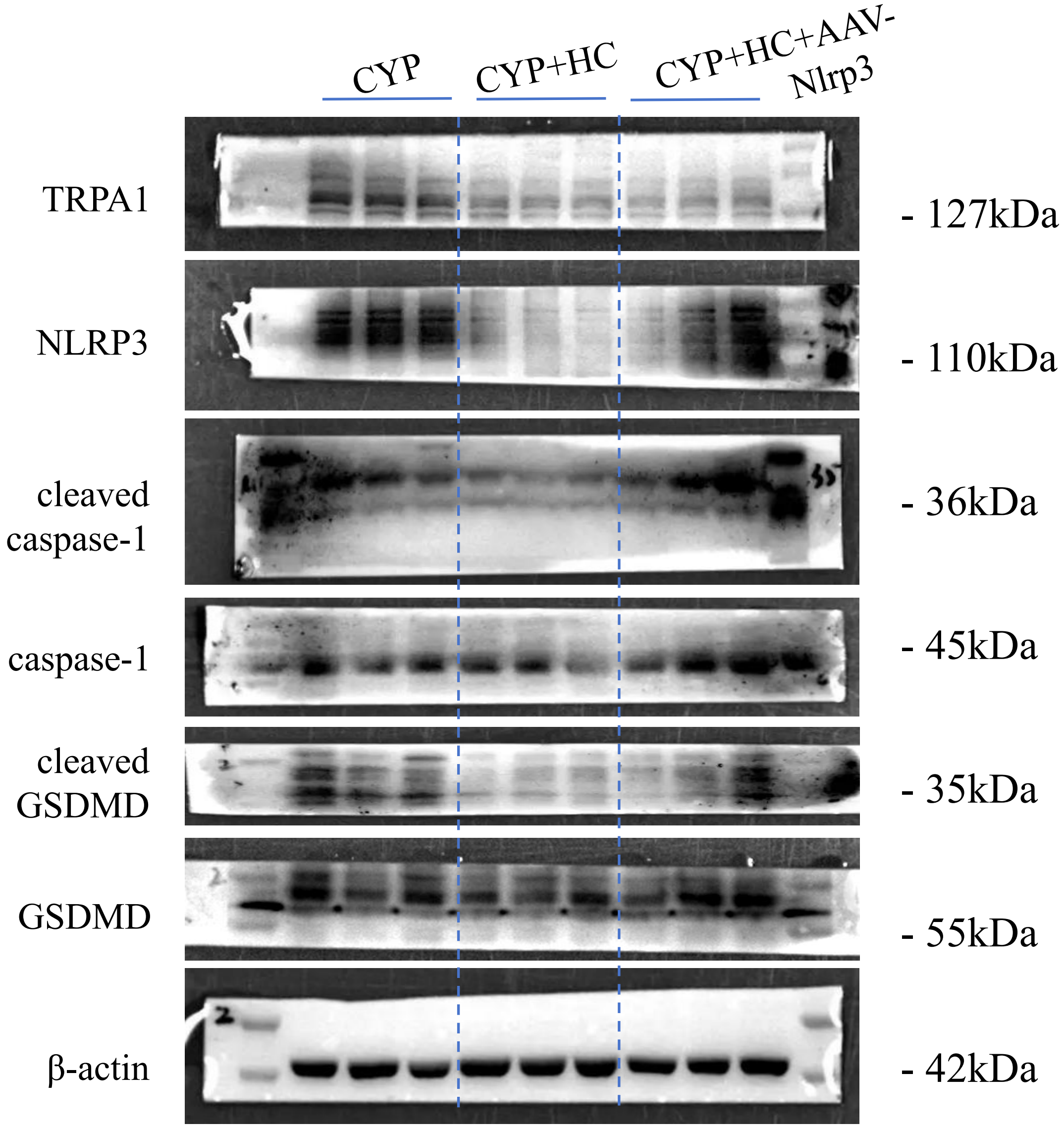

Figure 5E copy

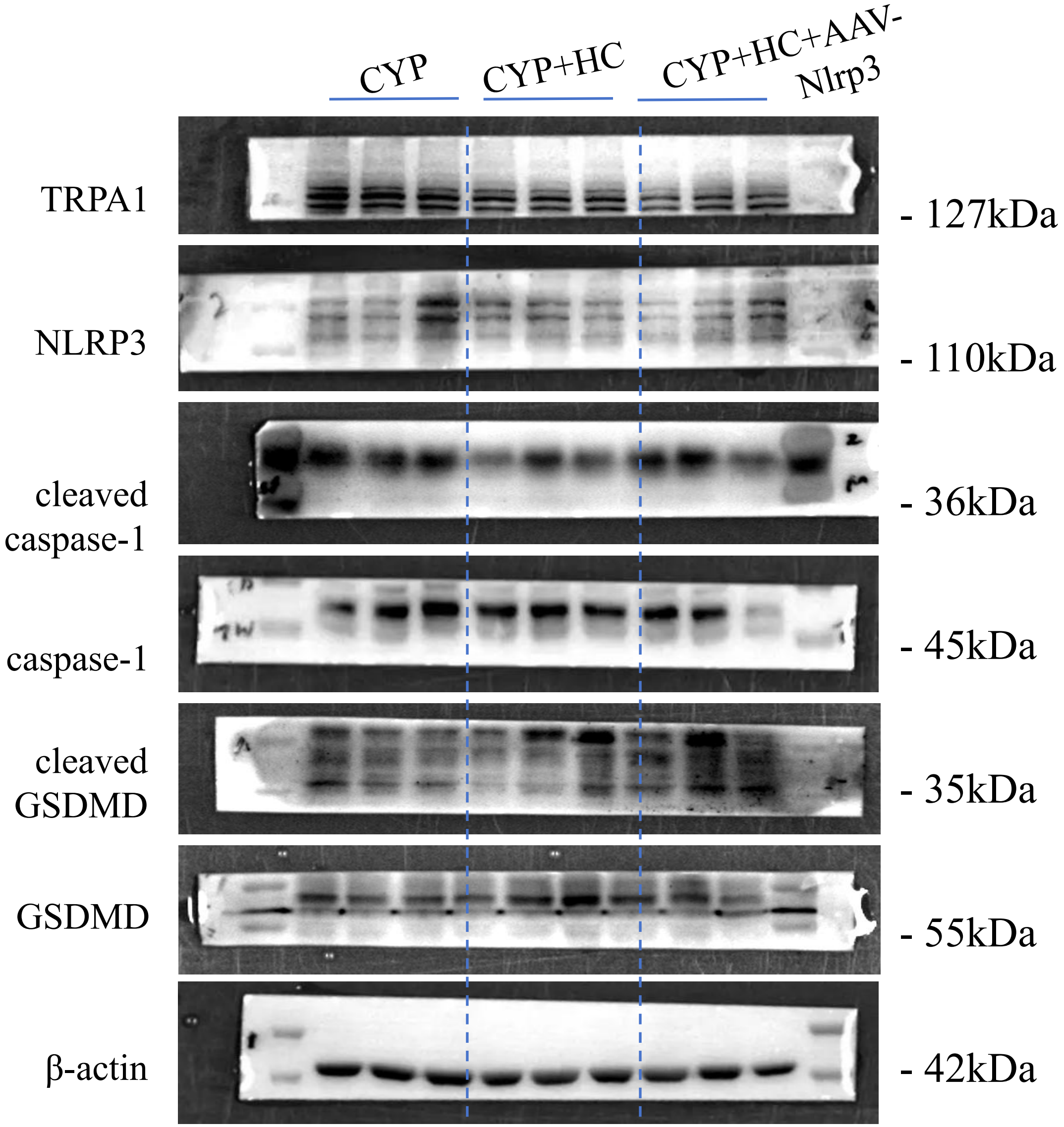

Full unedited gel for Figure 6F

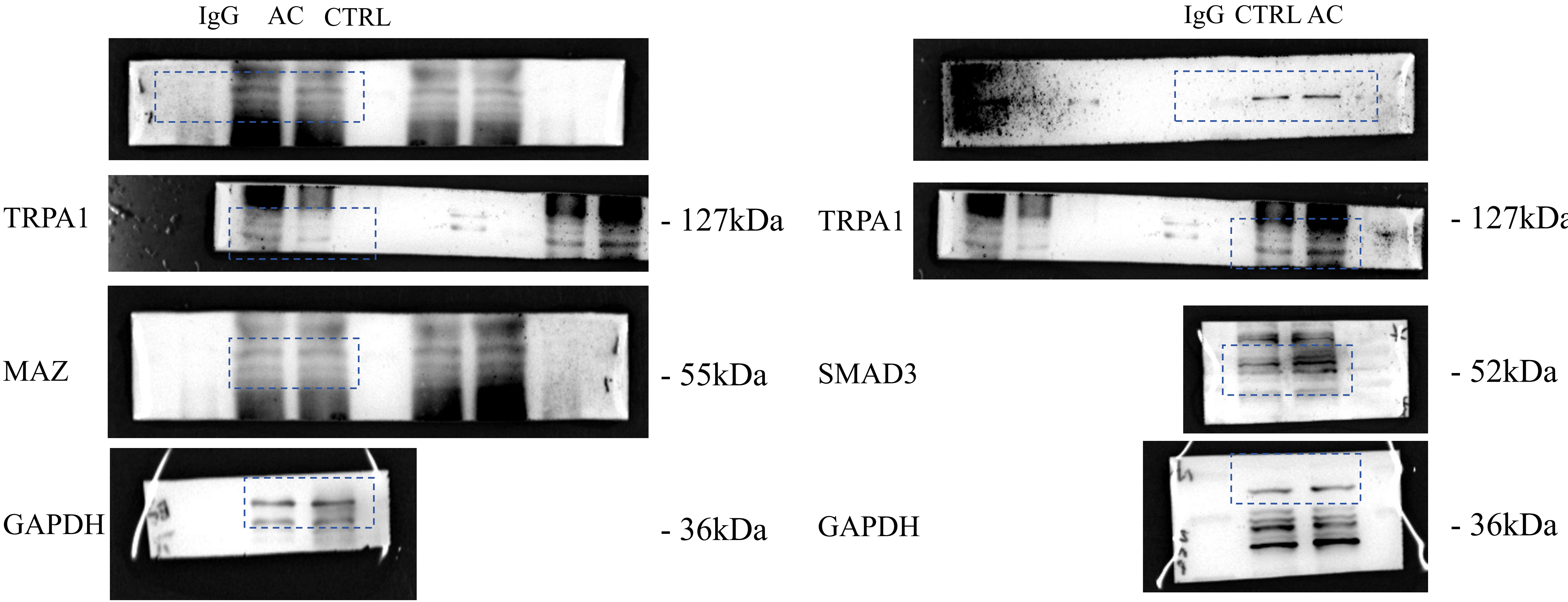

Full unedited gel for Figure 6H

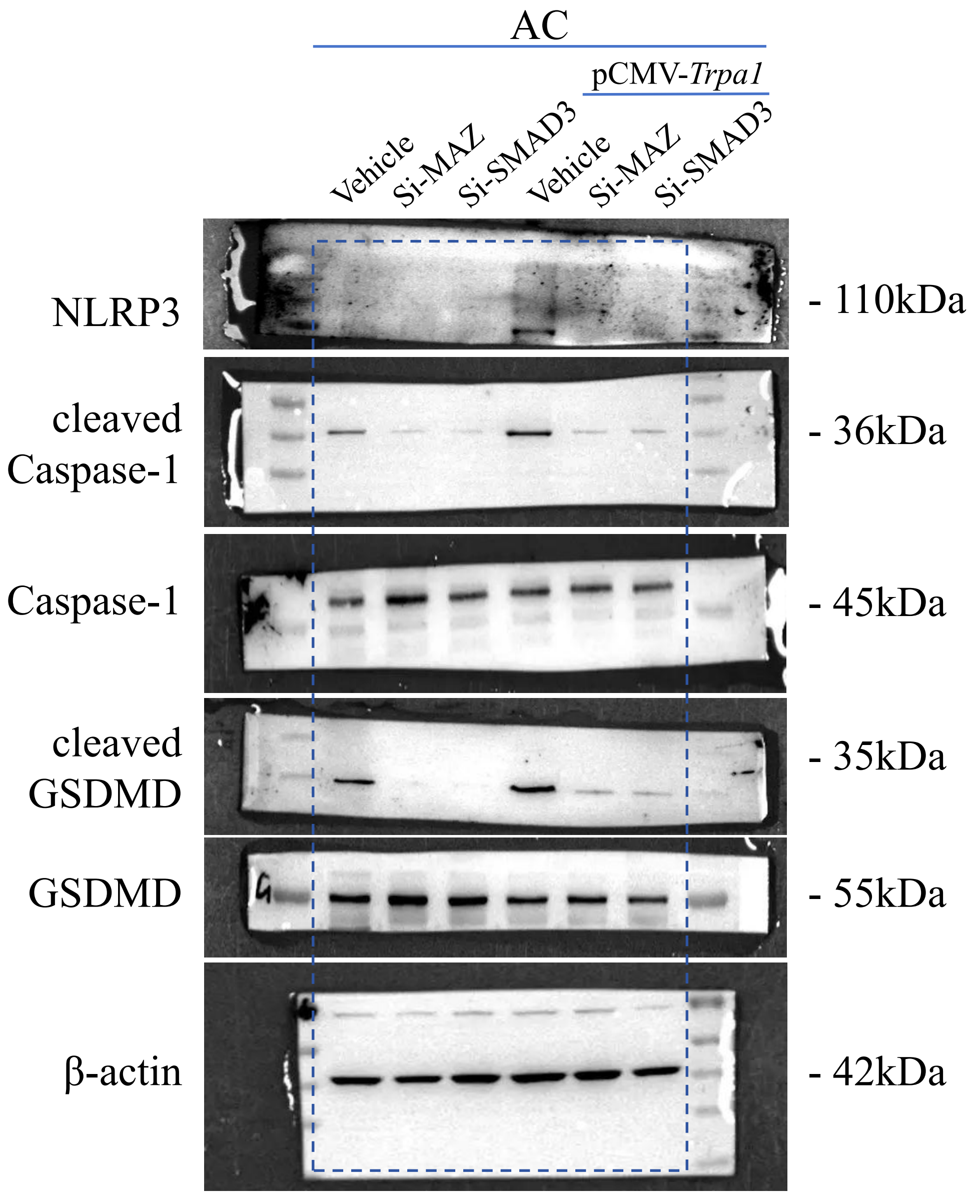

Figure 6H copy

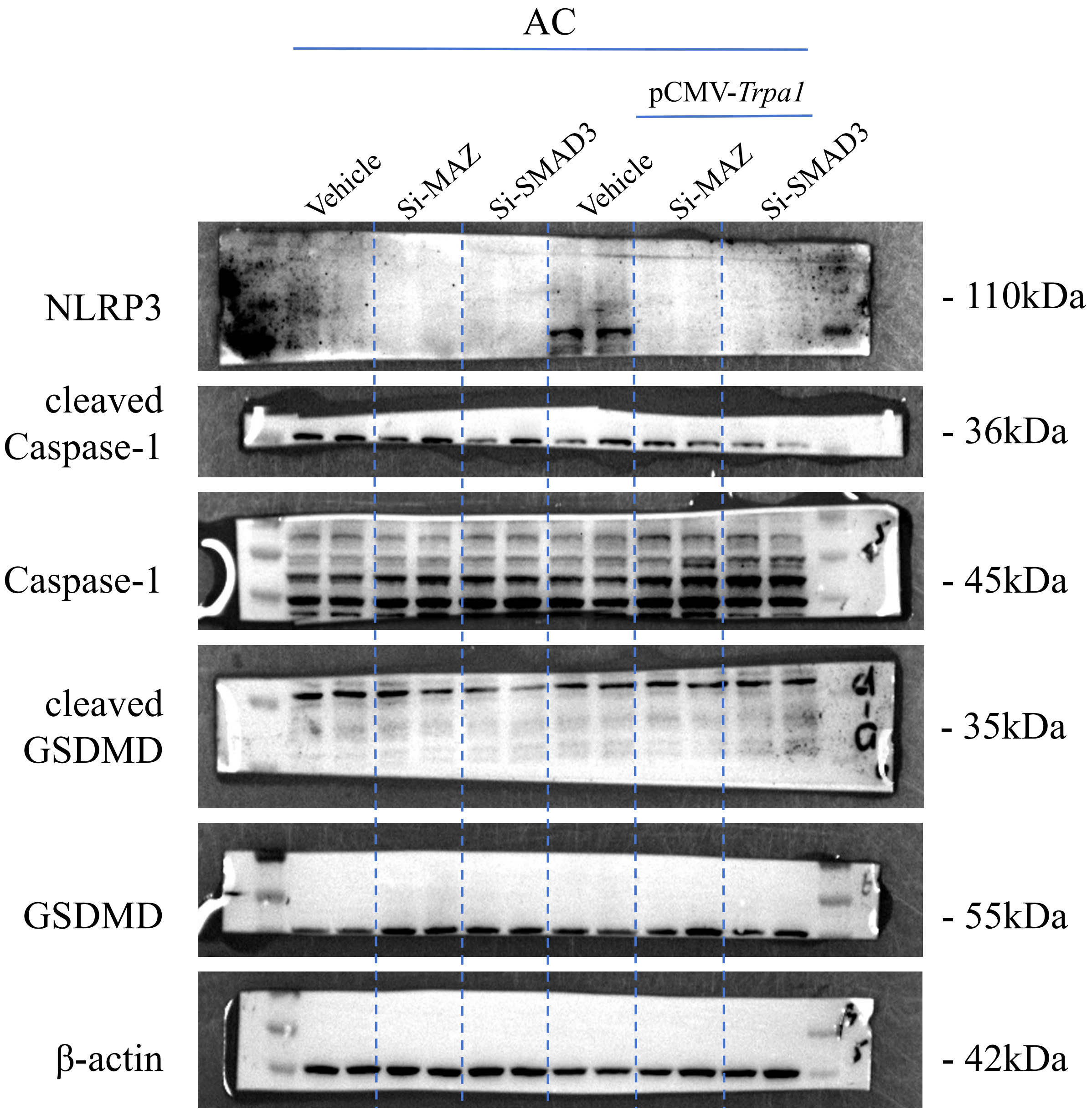

Supplement: Supplementary file 2 — Original Western blots [file 41419_2026_8426_MOESM2_ESM.pdf]
